# Supplementary material for: Hierarchical amplitude modulation structures and rhythm patterns: Comparing Western musical genres, song, and nature sounds to Babytalk
Source: PLoS One. 2022 Oct 14;17(10):e0275631. doi: 10.1371/journal.pone.0275631 (PMC9565671; doi:10.1371/journal.pone.0275631)
Supplement: S2 Appendix — (DOCX) [file pone.0275631.s002.docx]

*
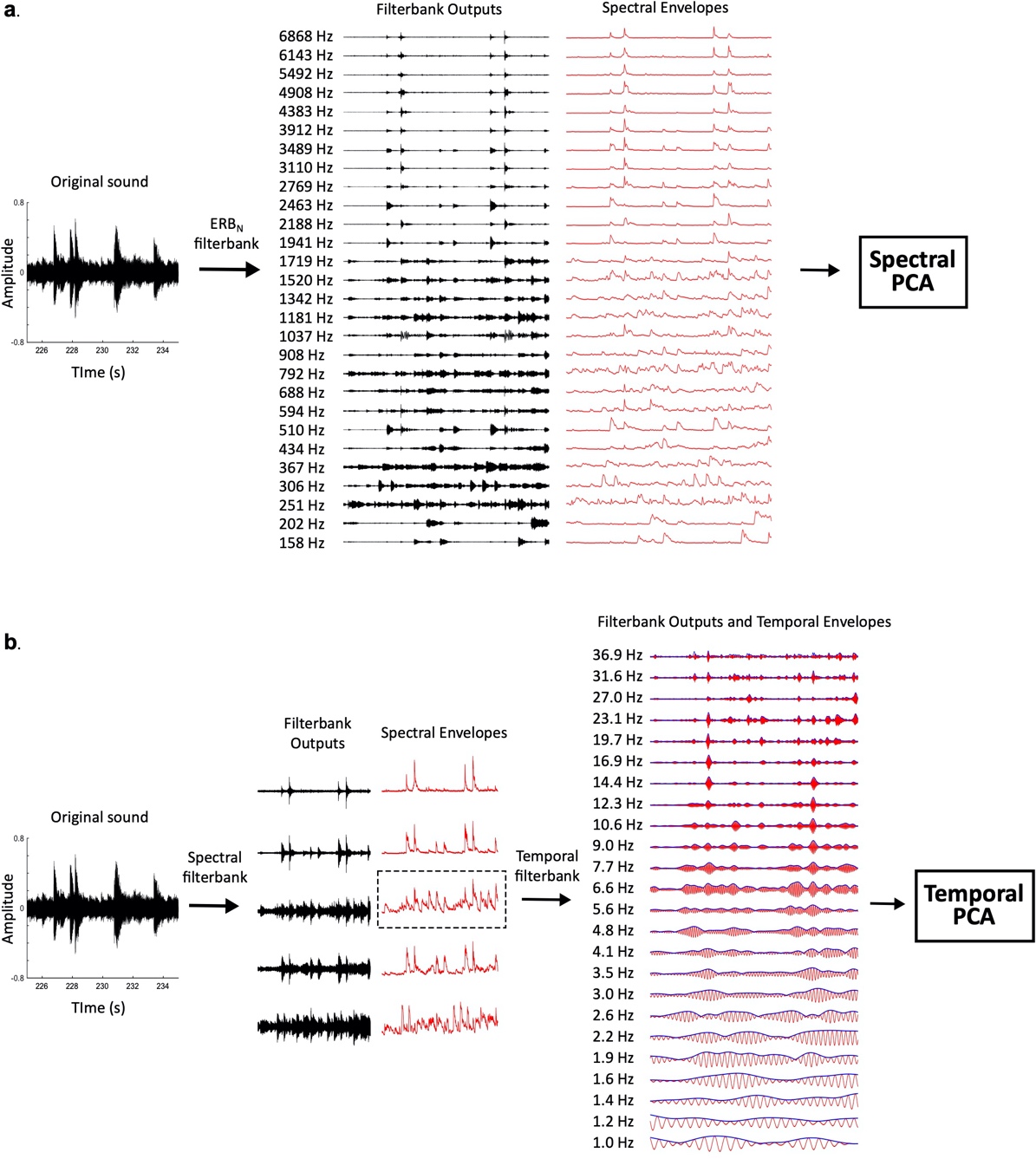
*

**Fig. a. Signal Processing Steps in S-AMPH Model.** (**a**) The original sound is a part of a sample used in this study (Beethoven Piano Sonata No.14, Op27 No 2 "Moonlight”). Original sound is passed through an ERB_N_-spaced filterbank, yielding a set of high-dimensional spectral channel outputs. The envelope is extracted from each spectral channel output using the Hilbert transform, and these envelopes are entered into the spectral PCA to identify patterns of covariation across spectral channels. (**b**) The original sound is passed through a low-dimensional spectral filterbank, yielding a small set of core spectral band outputs. The parameters of the low-dimensional spectral filterbank were determined in the Spectral PCA procedure (**a**). The envelopes are extracted from each spectral band output using the Hilbert transform. Each envelope is further passed through a high-dimensional modulation filterbank, yielding a set of high-dimensional modulation rate envelopes. This rate-filtering is performed for each spectral band envelope, but for simplicity, only the modulation rate envelopes from a single spectral band are shown in this figure. Finally, the power profiles of the modulation rate envelopes (bold blue line) are entered into a temporal PCA to identify patterns of covariation across modulation rates.

**Table. a. ERB filterbank (for 28 filter bank) and the frequency response characteristics.**

| Edge Number | Edge (Hz) |
| --- | --- |
| 1 (low-pass) | 100 |
| 2 | 137 |
| 3 | 179 |
| 4 | 225 |
| 5 | 277 |
| 6 | 334 |
| 7 | 398 |
| 8 | 470 |
| 9 | 549 |
| 10 | 638 |
| 11 | 736 |
| 12 | 846 |
| 13 | 969 |
| 14 | 1105 |
| 15 | 1257 |
| 16 | 1426 |
| 17 | 1614 |
| 18 | 1824 |
| 19 | 2057 |
| 20 | 2317 |
| 21 | 2607 |
| 22 | 2930 |
| 23 | 3289 |
| 24 | 3689 |
| 25 | 4135 |
| 26 | 4631 |
| 27 | 5184 |
| 28 | 5800 |
| 29 | 6486 |
| 30 | 7250 |

**Fig. b.** frequency response characteristics (28 filterbanks).

**Modulation filterbank (for 24 filter bank) and the frequency response characteristics (Fig. d and e).**

| Edge Number | Edge (Hz) |
| --- | --- |
| 1 (low-pass dummy channel,output discarded) | 0.79 |
| 2 | 0.93 |
| 3 | 1.09 |
| 4 | 1.27 |
| 5 | 1.49 |
| 6 | 1.74 |
| 7 | 2.03 |
| 8 | 2.38 |
| 9 | 2.78 |
| 10 | 3.25 |
| 11 | 3.80 |
| 12 | 4.45 |
| 13 | 5.20 |
| 14 | 6.08 |
| 15 | 7.11 |
| 16 | 8.32 |
| 17 | 9.72 |
| 18 | 11.38 |
| 19 | 13.30 |
| 20 | 15.56 |
| 21 | 18.20 |
| 22 | 21.28 |
| 23 | 24.89 |
| 24 | 29.11 |
| 25 | 34.04 |
| 26 | 39.81 |

**Fig. d.** frequency response characteristics (5 filterbanks).

**Fig. e.** frequency response characteristics (24 filterbanks).
