# Supplementary material for: Hierarchical amplitude modulation structures and rhythm patterns: Comparing Western musical genres, song, and nature sounds to Babytalk
Source: PLoS One. 2022 Oct 14;17(10):e0275631. doi: 10.1371/journal.pone.0275631 (PMC9565671; doi:10.1371/journal.pone.0275631)
Supplement: S3 Appendix — (DOCX) [file pone.0275631.s003.docx]

**Signal Processing Steps in PAD Model**

Example of an amplitude modulation (AM) hierarchy derived by recursive application of PAD. In the first demodulation round (left column), the data (**a**) are demodulated using PAD set to a fast time scale. This yields a relatively quickly-varying envelope (**b**) and a carrier (**c**). In the second demodulation round (middle column), the demodulation process is re-applied to the extracted envelope b, using a slower time scale than before. This yields a slower daughter envelope (**d**) and a faster daughter envelope (**e**). Daughter envelopes d and e form the two tiers of the resulting AM hierarchy (right column). Mathematically, these two tiers (**d** and **e**) can be multiplied back with the very first carrier (c, bottom left) to yield the original signal (**a**).

*
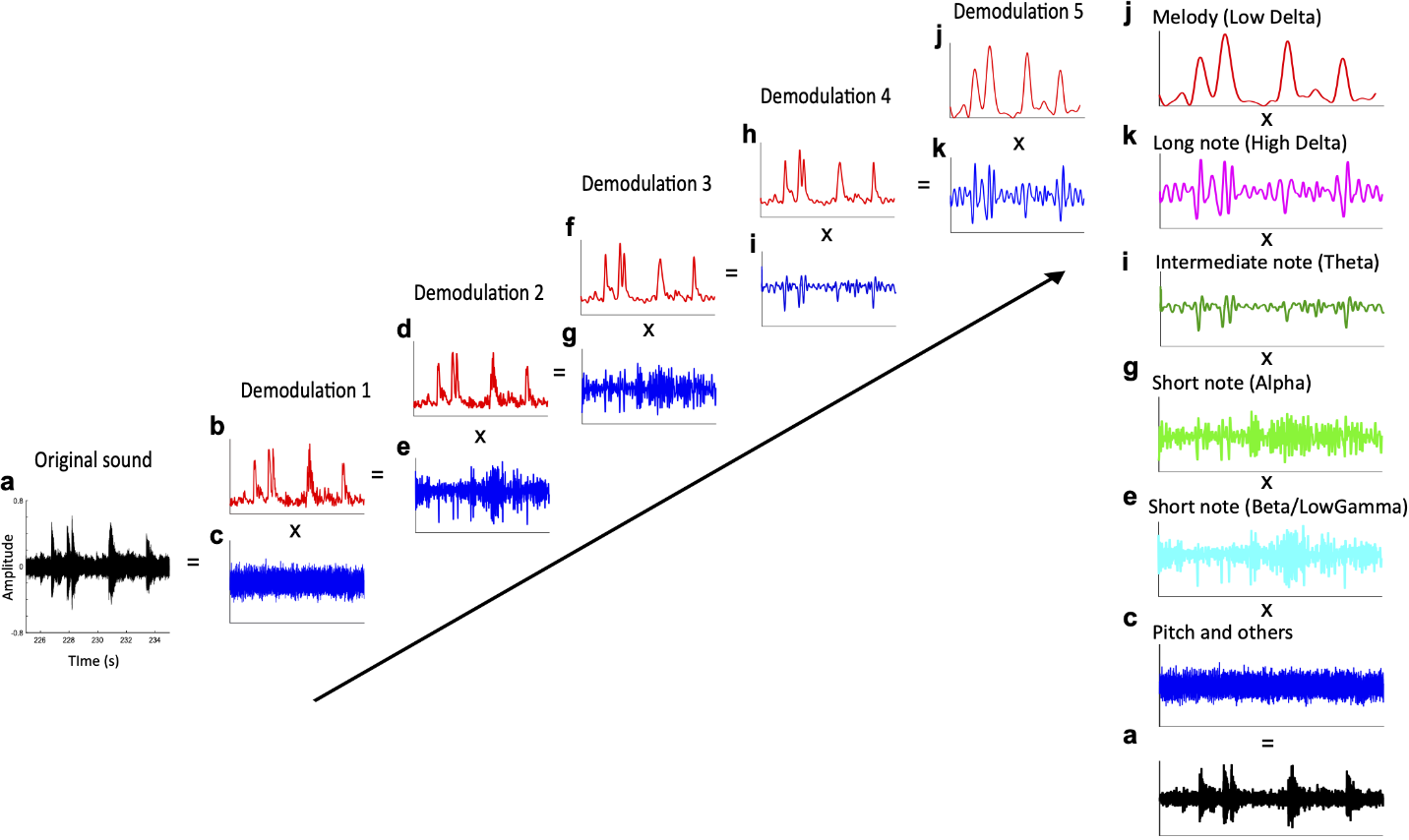
*
