# Supplementary material for: Hierarchical amplitude modulation structures and rhythm patterns: Comparing Western musical genres, song, and nature sounds to Babytalk
Source: PLoS One. 2022 Oct 14;17(10):e0275631. doi: 10.1371/journal.pone.0275631 (PMC9565671; doi:10.1371/journal.pone.0275631)
Supplement: S4 Appendix — (DOCX) [file pone.0275631.s004.docx]

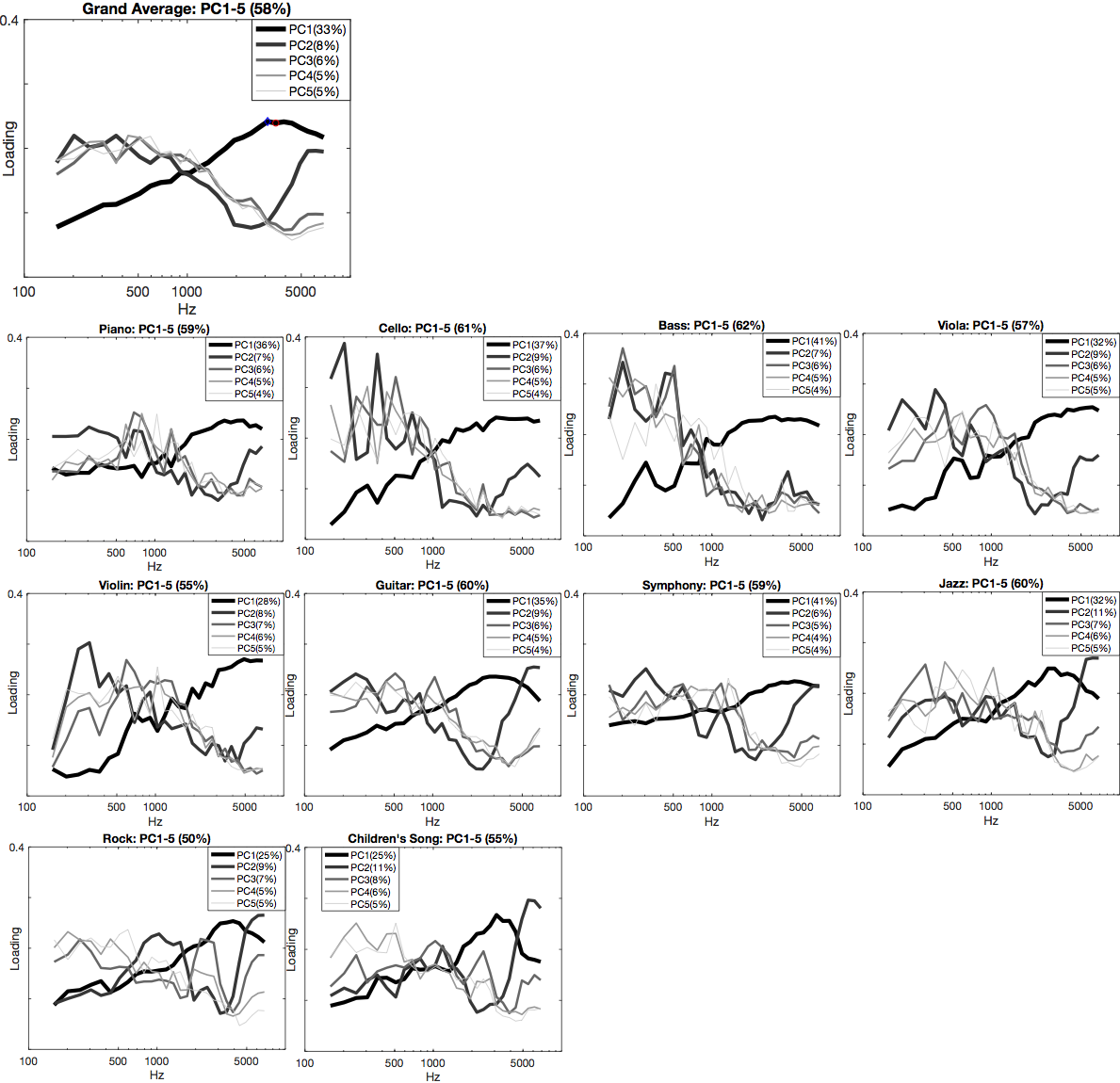


**Fig. a**. **Individual Variation in Spectral PCA Component Loadings and the cumulative accounts**

Figure **a** shows the spectral PCA component loading patterns for each genre. In each subplot, the lines of different thickness indicate different PCA components. More important (lower numbered) components are shown in a thicker line. The loading patterns for the top 5 PCA components are similar across the 10 genres. It may be observed that the 10 genres produced consistent PCA loading patterns, particularly for the first 3 components. We, however, also find the individual variation at each spectral modulation band.

The present study considered the core spectral bands using the grand average PC loading patterns. In the grand average, the first to fifth principal component (PC1 to PC5) accounted for, on average, 33%, 8%, 6%, 5%, and 5% of the total variances, respectively. One peak (~3000 Hz) was identified from the loading pattern of PC1. Thus, PC1 was assumed to reflect the global correlation between spectral channels. The peak of ~300 Hz and the “*flanking*” trough of ~350 Hz were identical between PC3 and PC4, providing corroborating evidence for a lowest spectral band at this spectral location with a potential boundary between the first and second spectral bands at ~350 Hz (troughs indicate potential boundaries between modulation rate bands). Further peaks and troughs were identified providing evidence for four further spectral bands.

In the end, a close peak of around 420-600 Hz and the identical flanking trough of ~700 Hz in PC3, PC4 and PC5, providing corroborating evidence for a second spectral band at this spectral location and a potential boundary between the second and third spectral bands at ~700 Hz. A close peak of 900-1100 Hz n PC3, PC4 and PC5, and a close flanking trough of 1750-2000 Hz in PC2, PC3 and PC5, provided corroborating evidence for a third spectral band at this spectral location and a potential boundary between the third and fourth spectral bands at ~1750 Hz. The peak of ~2500 Hz and the flanking trough of ~3900 Hz were identical between PC3 and PC5, providing corroborating evidence for a fourth spectral band at this spectral location and a potential boundary between the fourth and fifth spectral bands at ~3900 Hz. A peak of ~5500 Hz was identical between PC2 and PC3, providing corroborating evidence for a fifth spectral band at the spectral location. Further, this fifth peak was the highest peak observed in the spectral PCA loading patterns.

**Table a. Summary of the 5 spectral bands and the 4 flanking boundaries indentified from spectral PCA.**

| **Spectral bands** | **Frequency range (Hz)** | **PC Peaks** |
| --- | --- | --- |
| Band 1 | 100-350 | PC2-PC5 |
| Band 2 | 350-700 | PC2-PC5 |
| Band 3 | 700-1750 | PC3-PC5 |
| Band 4 | 1750-3900 | PC1, PC3, PC5 |
| Band 5 | 3900-7250 | PC2, PC3 |


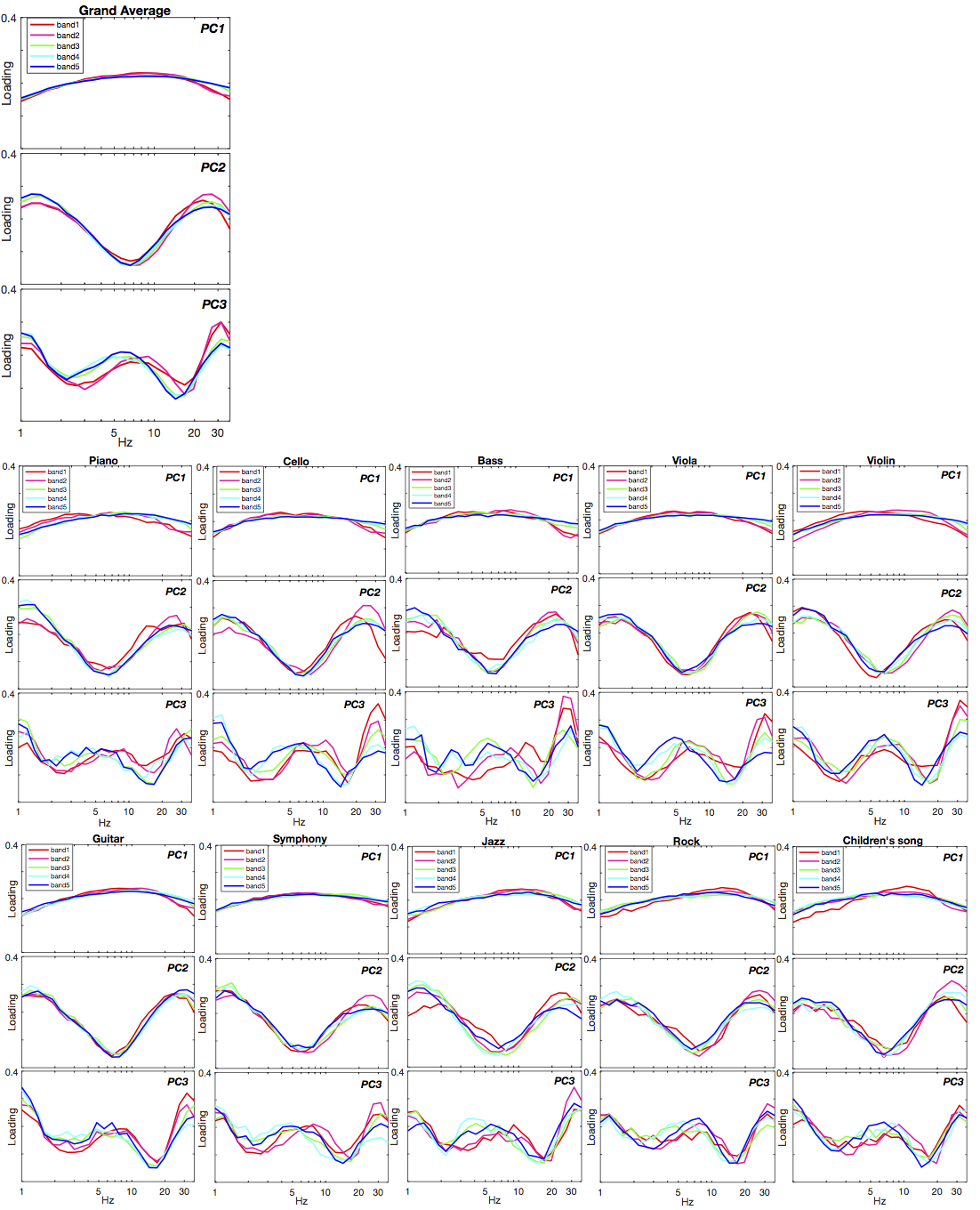


**Fig. b. Individual Variation in Temporal PCA Component Loadings**. Fig. **b** shows the temporal PCA component loading patterns for each genre. In each subplot, the lines of different thickness indicate different PCA components. More important (lower numbered) components are shown in a thicker line, and the components from the 5 spectral bands are plotted in different colours. It may be observed that the 10 genres produced consistent PCA loading patterns. We, however, also find the individual variation at each spectral modulation band.

The present study considered the core temporal bands using the grand average PC loading patterns. In the grand average, the first to third principal component (PC1 to PC3) accounted for, on average, 49%, 11% and 6% of the total variances, respectively. PC1 showed a moderate peak at acoustic frequencies of 7-9 Hz in all of the 5 spectral bands. As observed in the spectral PCA, PC1 in the temporal PCA might reflect the global correlation between temporal channels. As no troughs were detected in PC1 (indicating no potential boundaries), our analysis focused on PC2 and PC3. The loading patterns of PC2 resulted in 2 strong peaks at acoustic frequencies of 1-2 Hz (evidence for a delta-rate band of AMs) and 20-30 Hz (evidence for a beta-gamma rate band of AMs), and 1 strong flanking trough at acoustic frequencies of ~7 Hz. These findings were consistent between the 5 spectral bands, suggesting the potential existence of at least 2 core temporal bands. Compared with PC1 and PC2, PC3 loading patterns varied across spectral bands. As detected in PC2, all the spectral bands showed a peak in loading at ~30 Hz, and spectral band 2 similarly showed a peak at ~1 Hz. PC3 also showed an additional mid-rate peak at ~5 Hz (theta-rate band) and ~9 Hz (alpha-rate band). The flanking troughs for these peaks occurred 2-3 Hz and 15-18 Hz. Based on the a priori criteria (Methods), the temporal PCA thus provided evidence for the presence of 4 core bands with 3 boundaries across the different musical genres and instruments. Perceptually, cycles in these AM bands may yield the experience of crotchets, quavers, demiquavers and onsets, as shown in Table 1 of the manuscript.

**Table b. Cumulative accounts of temporal PCA of 5 bands indentified from spectral PCA.**

| **Genre** | **PC** | **band1** | **band2** | **band3** | **band4** | **band5** | **mean** |
| --- | --- | --- | --- | --- | --- | --- | --- |
| Grand average | PC1 | 0.43 | 0.44 | 0.48 | 0.52 | 0.56 | 0.486 |
| Piano | PC2 | 0.11 | 0.11 | 0.10 | 0.10 | 0.11 | 0.106 |
|  | PC3 | 0.07 | 0.07 | 0.06 | 0.05 | 0.05 | 0.06 |
|  | PC1 | 0.44 | 0.45 | 0.55 | 0.58 | 0.64 | 0.532 |
|  | PC2 | 0.12 | 0.11 | 0.11 | 0.11 | 0.12 | 0.114 |
|  | PC3 | 0.07 | 0.07 | 0.06 | 0.05 | 0.05 | 0.06 |
| Cello | PC1 | 0.40 | 0.42 | 0.46 | 0.53 | 0.58 | 0.478 |
|  | PC2 | 0.11 | 0.11 | 0.09 | 0.09 | 0.09 | 0.098 |
|  | PC3 | 0.07 | 0.07 | 0.06 | 0.05 | 0.05 | 0.06 |
| Bass | PC1 | 0.38 | 0.39 | 0.49 | 0.53 | 0.58 | 0.474 |
|  | PC2 | 0.11 | 0.12 | 0.11 | 0.09 | 0.09 | 0.104 |
|  | PC3 | 0.08 | 0.07 | 0.06 | 0.05 | 0.05 | 0.062 |
| Viola | PC1 | 0.48 | 0.47 | 0.49 | 0.53 | 0.59 | 0.512 |
|  | PC2 | 0.12 | 0.12 | 0.10 | 0.09 | 0.09 | 0.104 |
|  | PC3 | 0.06 | 0.06 | 0.05 | 0.05 | 0.05 | 0.054 |
| Violin | PC1 | 0.54 | 0.42 | 0.47 | 0.49 | 0.54 | 0.492 |
|  | PC2 | 0.13 | 0.12 | 0.10 | 0.10 | 0.10 | 0.11 |
|  | PC3 | 0.06 | 0.07 | 0.06 | 0.06 | 0.05 | 0.06 |
| Guitar | PC1 | 0.49 | 0.48 | 0.51 | 0.56 | 0.62 | 0.532 |
|  | PC2 | 0.12 | 0.11 | 0.11 | 0.11 | 0.13 | 0.116 |
|  | PC3 | 0.07 | 0.06 | 0.06 | 0.05 | 0.05 | 0.058 |
| Synphony | PC1 | 0.48 | 0.46 | 0.48 | 0.52 | 0.55 | 0.498 |
|  | PC2 | 0.10 | 0.10 | 0.09 | 0.09 | 0.10 | 0.096 |
|  | PC3 | 0.05 | 0.06 | 0.05 | 0.05 | 0.05 | 0.052 |
| Jazz | PC1 | 0.42 | 0.45 | 0.53 | 0.55 | 0.52 | 0.494 |
|  | PC2 | 0.12 | 0.11 | 0.12 | 0.11 | 0.12 | 0.116 |
|  | PC3 | 0.07 | 0.06 | 0.05 | 0.05 | 0.06 | 0.058 |
| Rock | PC1 | 0.32 | 0.38 | 0.37 | 0.38 | 0.44 | 0.378 |
|  | PC2 | 0.11 | 0.11 | 0.10 | 0.10 | 0.14 | 0.112 |
|  | PC3 | 0.07 | 0.06 | 0.06 | 0.06 | 0.07 | 0.064 |
| Children's song | PC1 | 0.37 | 0.49 | 0.47 | 0.51 | 0.53 | 0.474 |
|  | PC2 | 0.11 | 0.11 | 0.12 | 0.12 | 0.15 | 0.122 |
|  | PC3 | 0.08 | 0.06 | 0.07 | 0.06 | 0.06 | 0.066 |

**Table c. Summary of the 4 temporal bands and the 3 flanking boundaries indentified from temporal PCA.**

Using an ERB_N_ filterbank that simulated the frequency decomposition by the cochlea, the S-AMPH model generated an hierarchical representation of the core spectral (acoustic frequency spanning 100–7,250 Hz) and temporal (oscillatory rate spanning 0.9–40 Hz) modulation architectures in the envelope of a range of musical genres and instruments. In each genre and for each instrument, the AM hierarchies consisted of 5 spectral bands (100–350 Hz; 350–700 Hz; 700–1750 Hz; 1750–3900 Hz; 3900–7250 Hz), each containing 4 hierarchically-nested temporal bands (0.9–2.5 Hz, 2.5–7 Hz, 7–17 Hz, 17–40 Hz).

| **Temporal bands** | **Frequency range (Hz)** | **PC Peaks** |
| --- | --- | --- |
| Band 1 | 0.9-2.5 | PC2, PC3 in spectral band 1-5 |
| Band 2 | 2.5-7 | PC3 in spectral band 3-5 |
| Band 3 | 7-17 | PC3 in spectral band 1-2 |
| Band 4 | 17-40 | PC2, PC3 in spectral band 1-5 |

**Fig. d. Individual modulation spectra of FFT. Individual modulation spectra of FFT in the PAD model** Fig. **d** shows the modulation spectra of FFT for each genre. The 10 genres are plotted in different colours. It may be observed that the 10 genres produced broadly consistent modulation spectra of FFT.

*
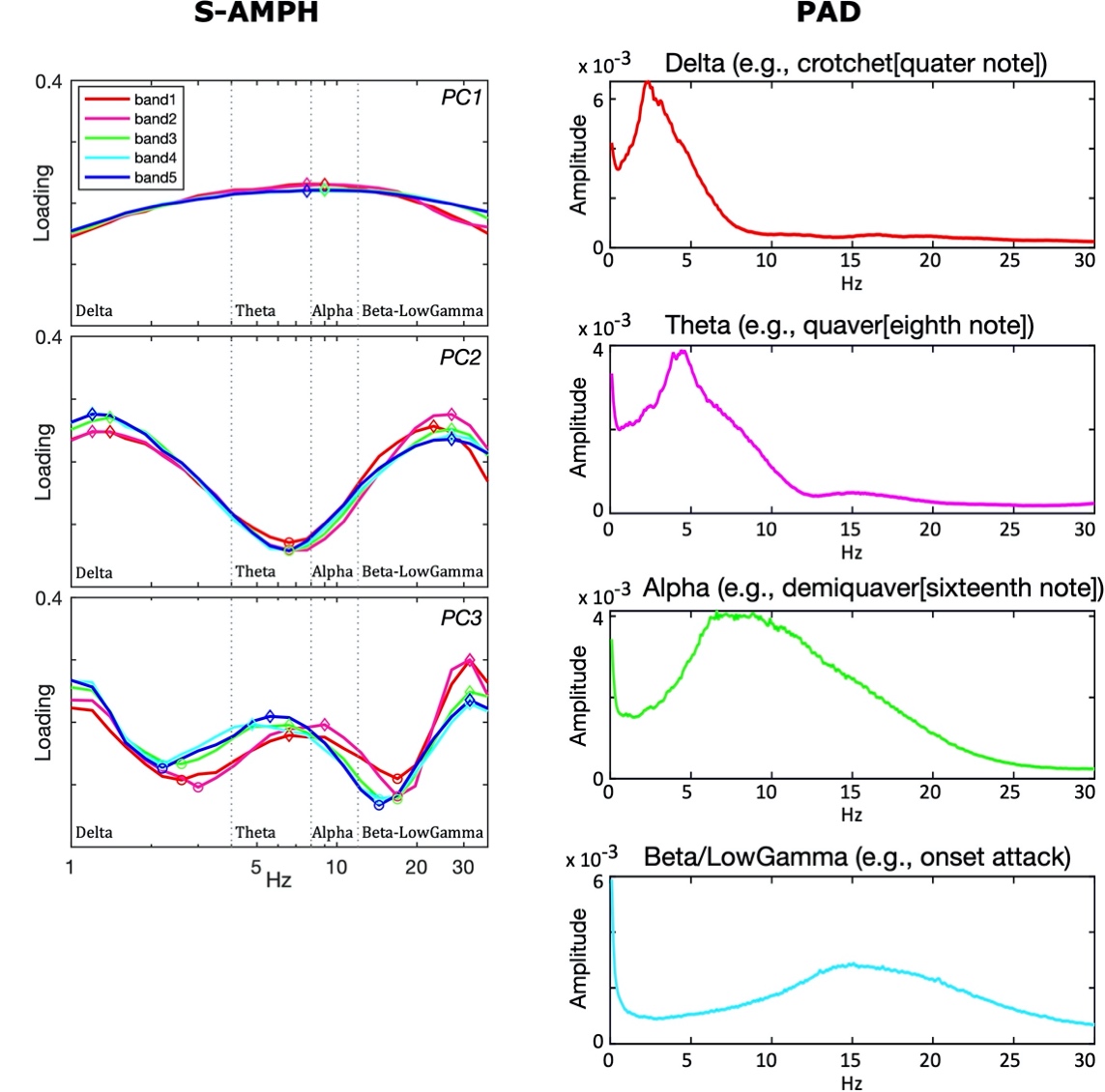
*

**Fig. e. Core Temporal Modulation Rates of SD-AMPH.** Grand average absolute value of temporal PCA component loading patterns in the S-AMPH (a) model. Both models showed an amplitude modulations (AM) hierarchy that consisted of delta-, theta-, alpha- and beta-rate AM bands. The colors represent the 5 spectral bands derived by the modelling, while the types of lines (i.e., bold, dashed and dotted) represent PCs 1,2 and 3, respectively. As may be clearly observed, the loading showed consistent patterns between the 3 PCA loading patterns. The detailed results were described from the next page.

**Table d.**

*Summary of the 4 Temporal Bands in Western Music Identified from PCA by Both S-AMPH and PAD*

| Temporal bands | S-AMPH | PAD | Neural Oscillatory Rates | Note value |
| --- | --- | --- | --- | --- |
| Band 1 | 0.9-2.5 Hz | -4 Hz | Delta | Crotchet (quarter note) |
| Band 2 | 2.5-7 Hz | 4-8 Hz | Theta | Quaver (eighth note) |
| Band 3 | 7-17 Hz | 8-12 Hz | Alpha | Demiquaver (sixteenth note) |
| Band 4 | 17-40 Hz | 12-30 Hz | Beta/LowGamma | Onset attack |
