# Supplementary material for: Hierarchical amplitude modulation structures and rhythm patterns: Comparing Western musical genres, song, and nature sounds to Babytalk
Source: PLoS One. 2022 Oct 14;17(10):e0275631. doi: 10.1371/journal.pone.0275631 (PMC9565671; doi:10.1371/journal.pone.0275631)
Supplement: S5 Appendix — (DOCX) [file pone.0275631.s005.docx]

**Grand average of mutual Information in S-AMPH model (averaged different Bands).**

Both S-AMPH (a) and PAD models (b) showed stronger dependency between each adjacent tier of an amplitude modulations (AM) hierarchy. Further, mutual dependence between delta- and theta-rate AM bands was the strongest of all mutual dependence in both models.

*
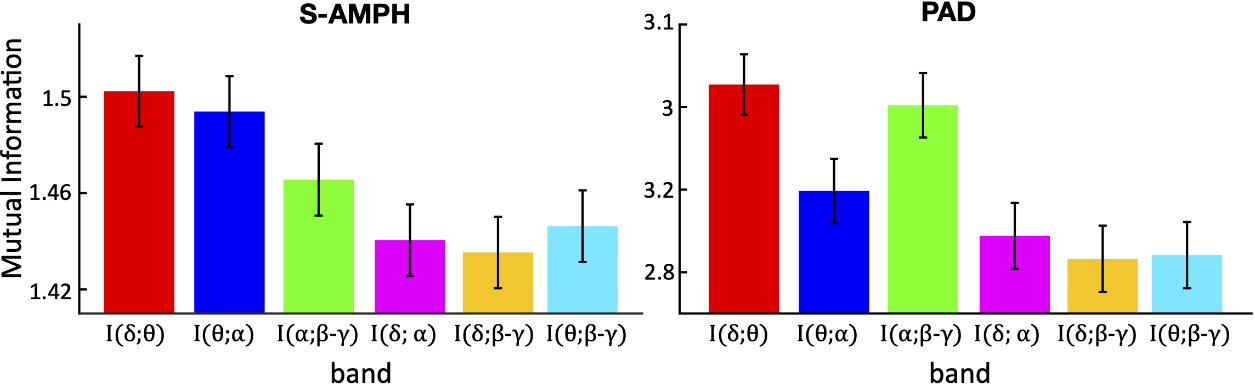
*

**Mutual Information of each genre Between different Bands in S-AMPH model.**

Figure **a** shows the mutual information (MI) between different bands in S-AMPH model for each genre. The MI shown here are broadly similar across the 10 genres. Figure **b** shows the MI of non-music natural sounds such as rain and wind.

**Figure a**. Mutual information between different bands in S-AMPH model

**
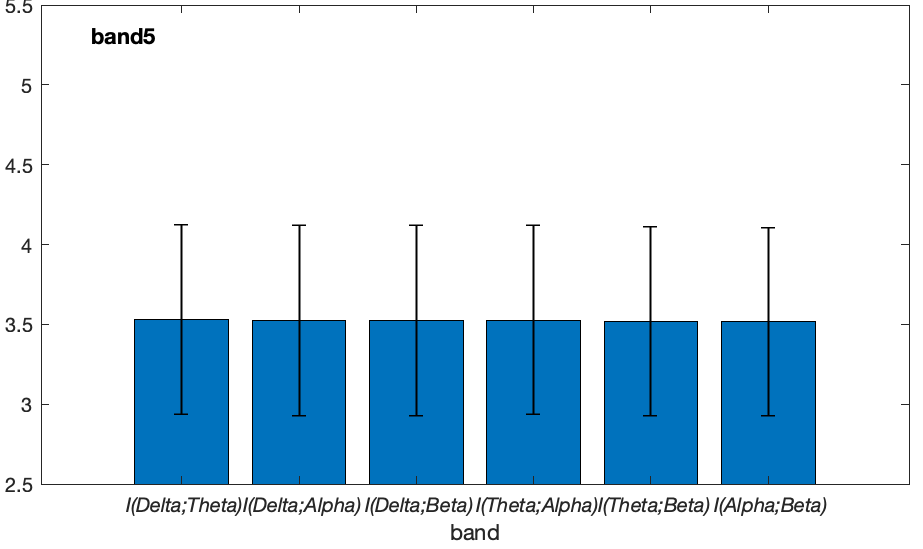

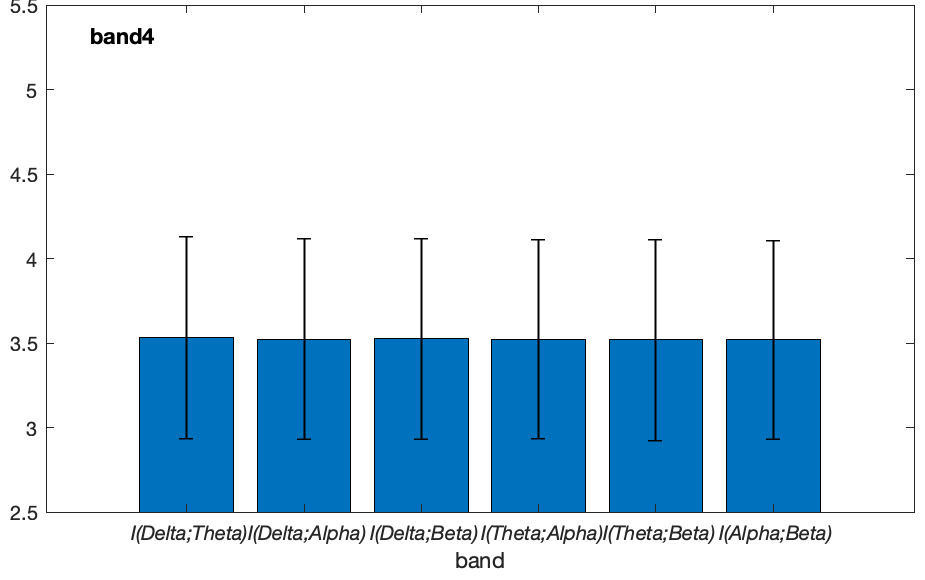

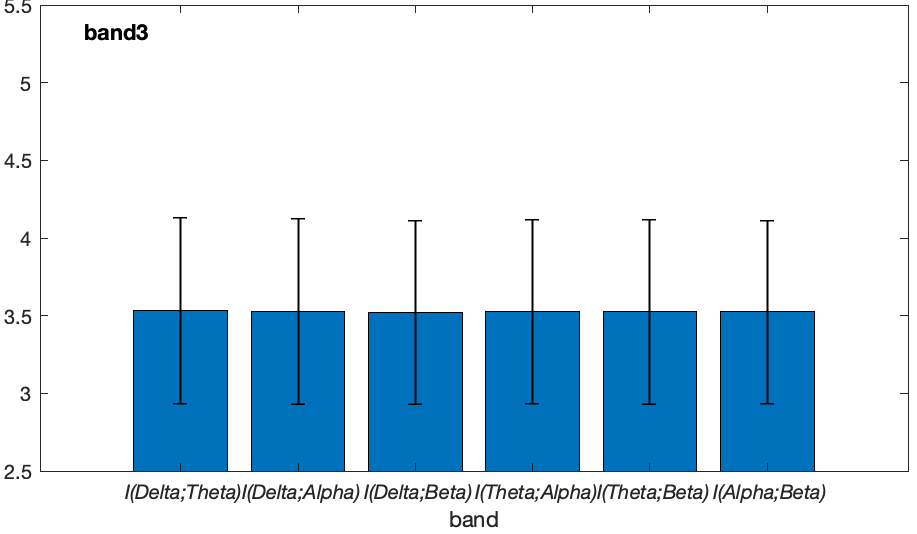

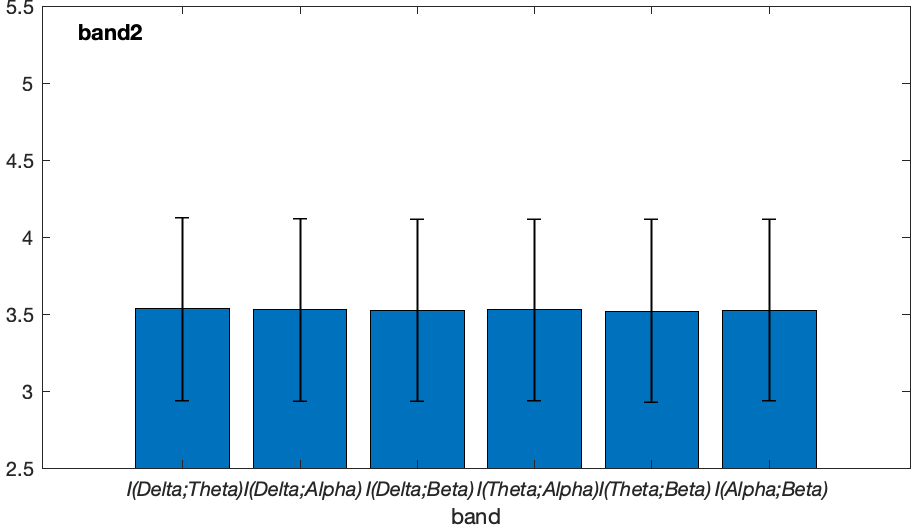

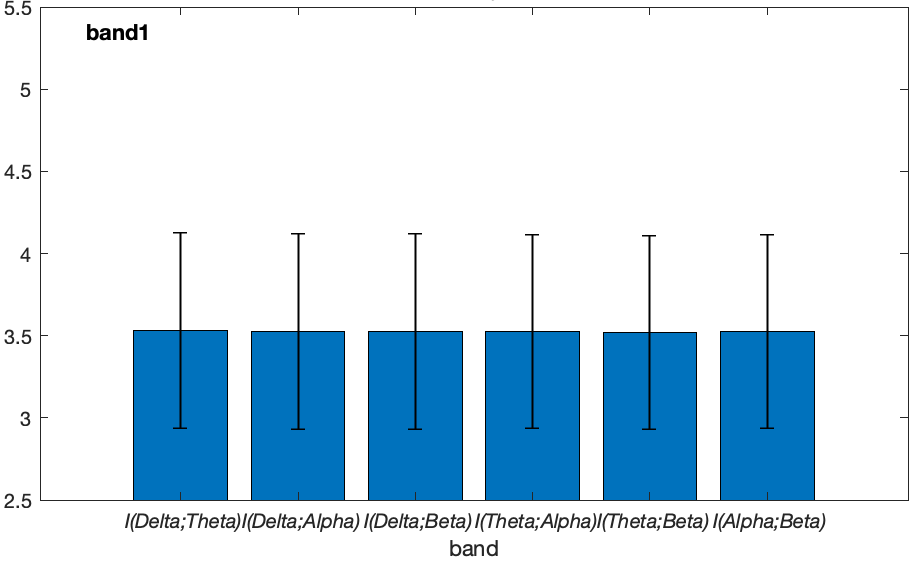

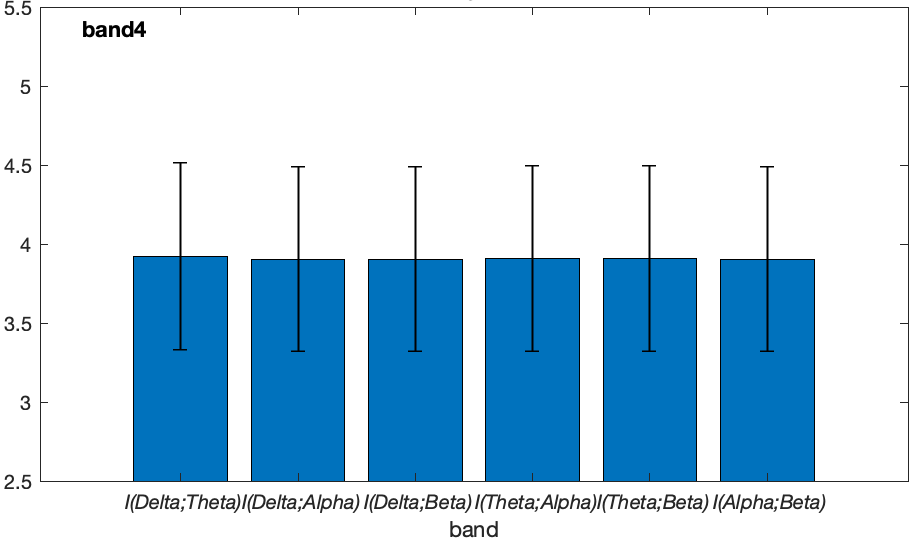

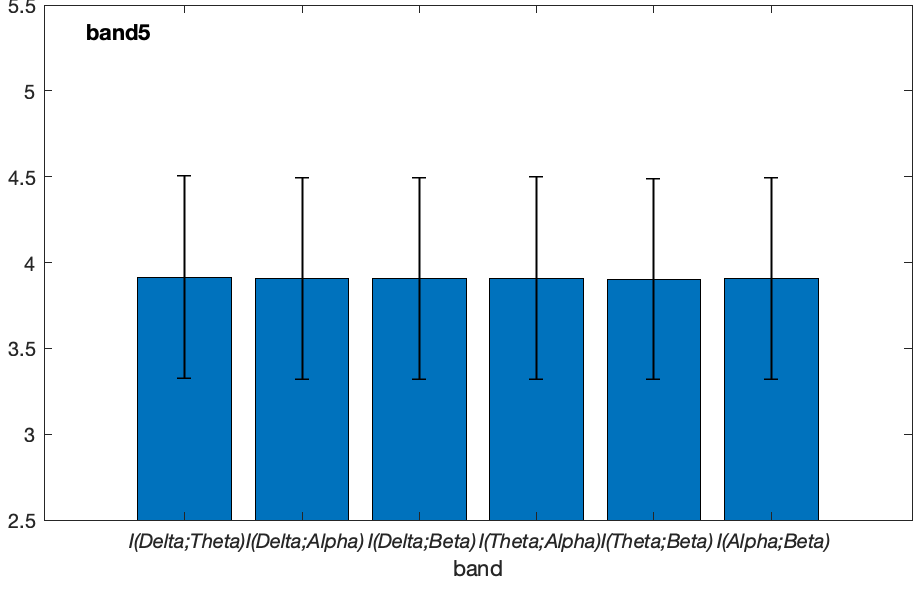

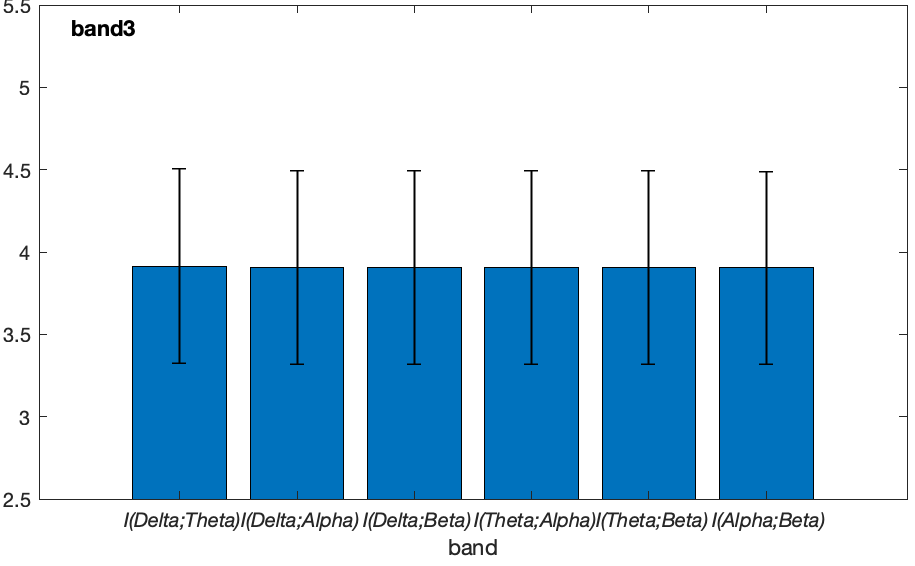

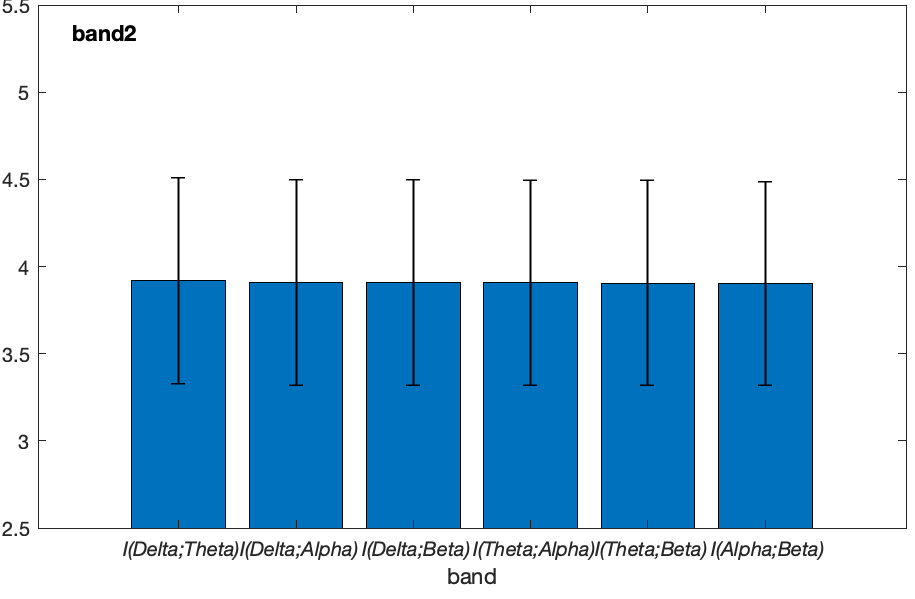

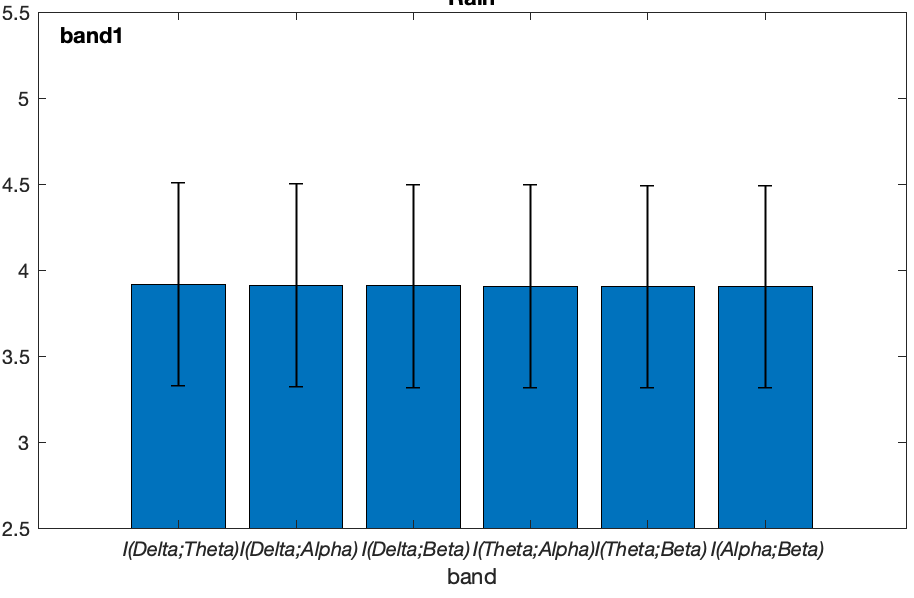

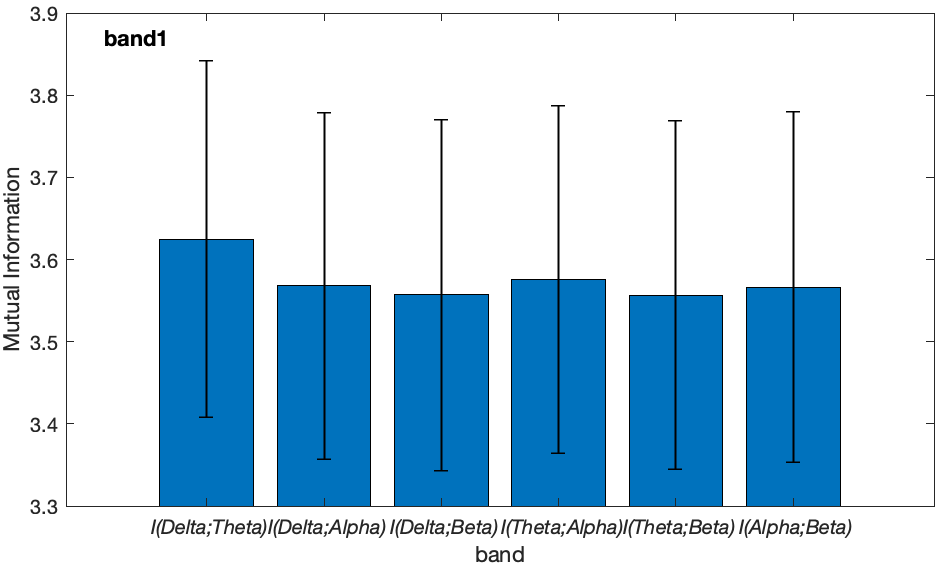

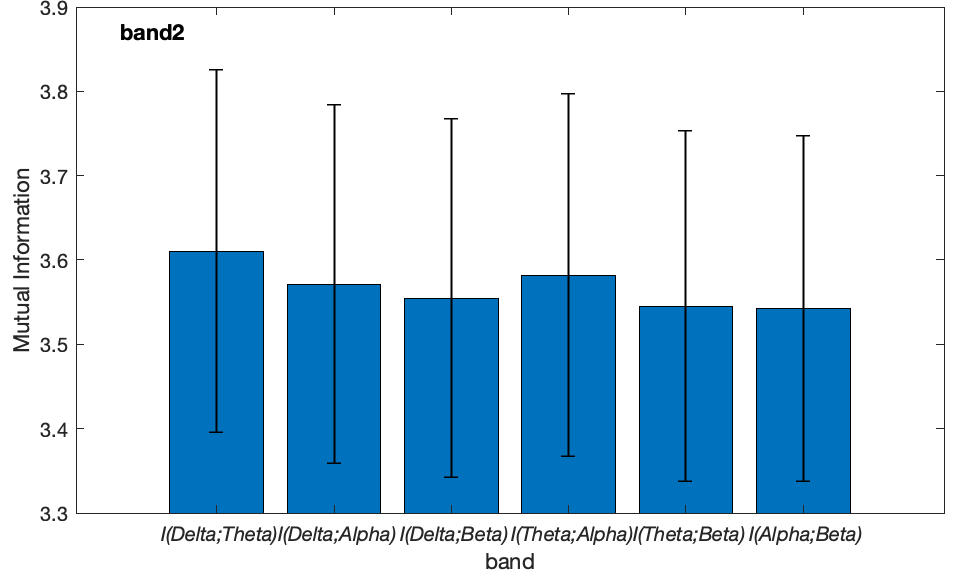

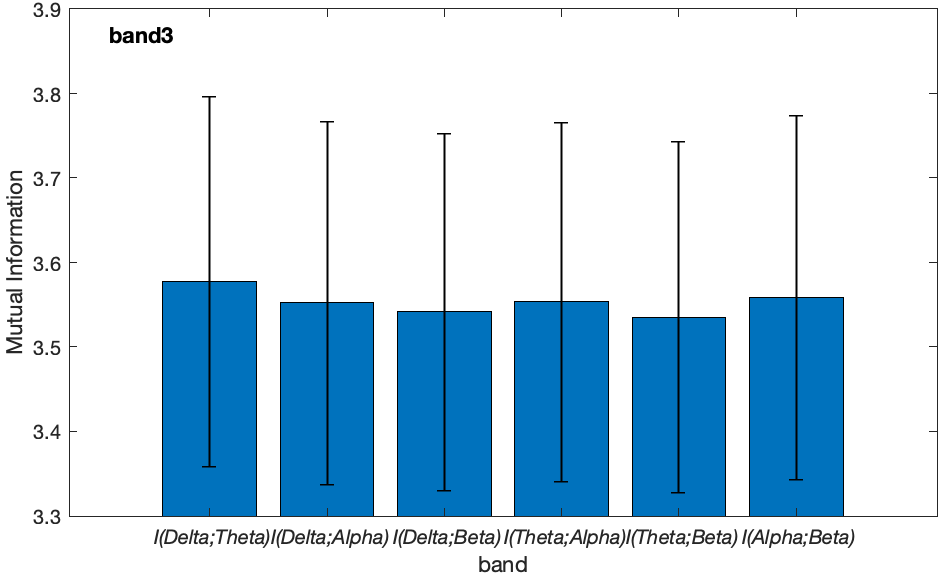

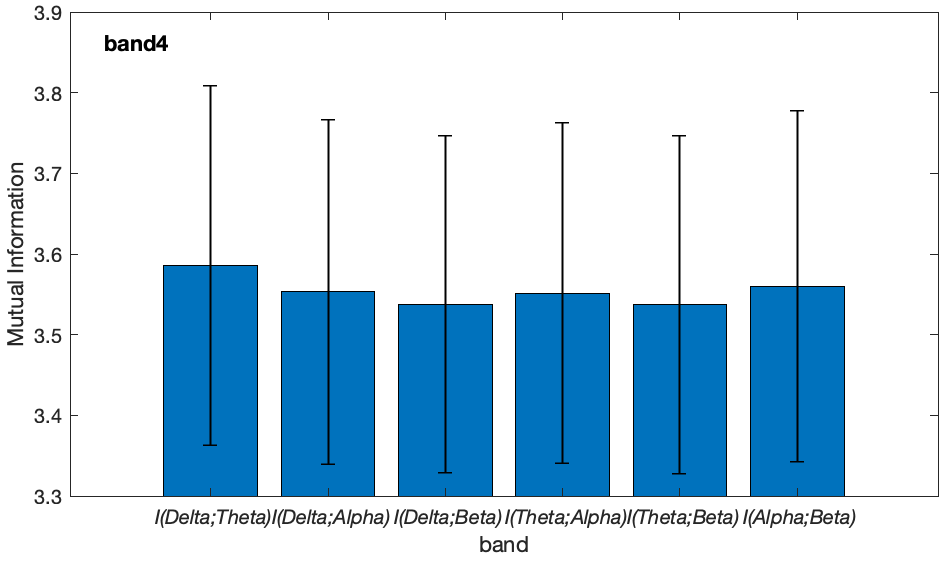

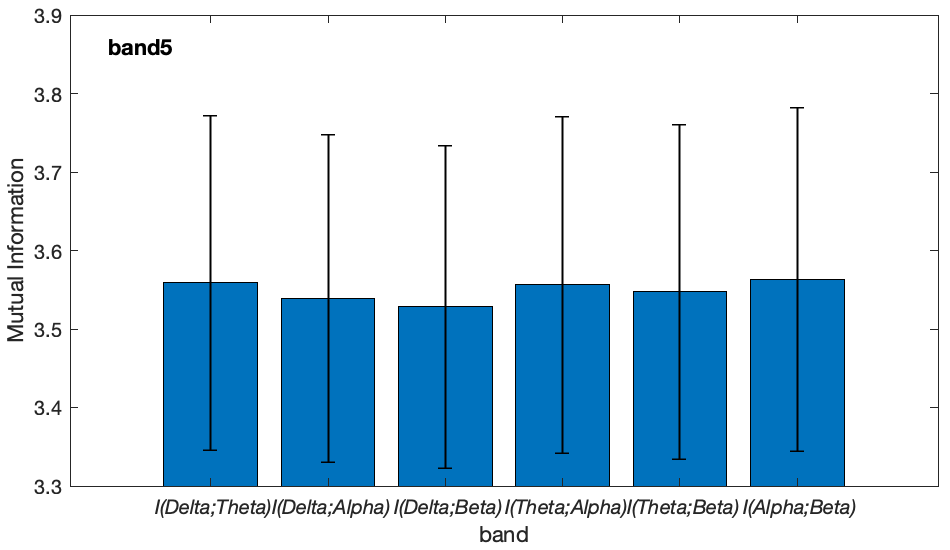

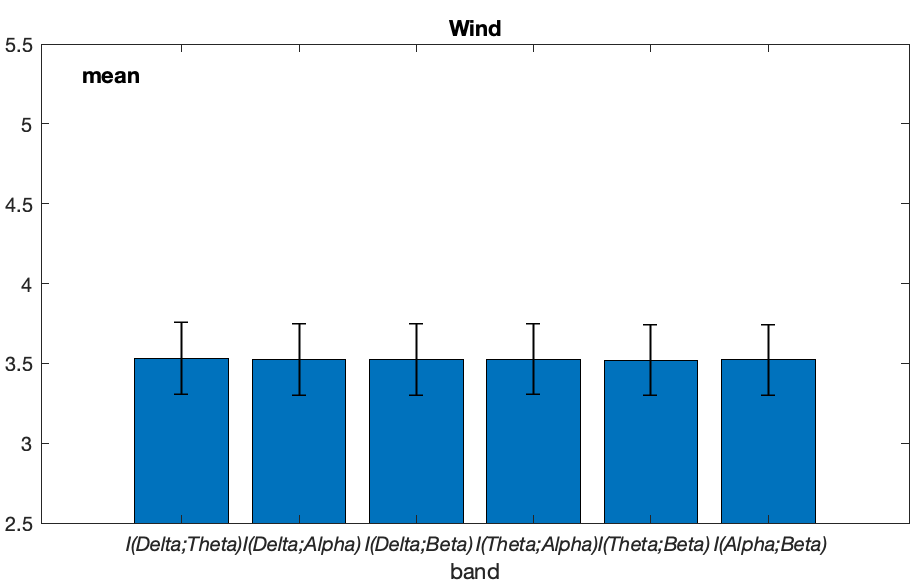

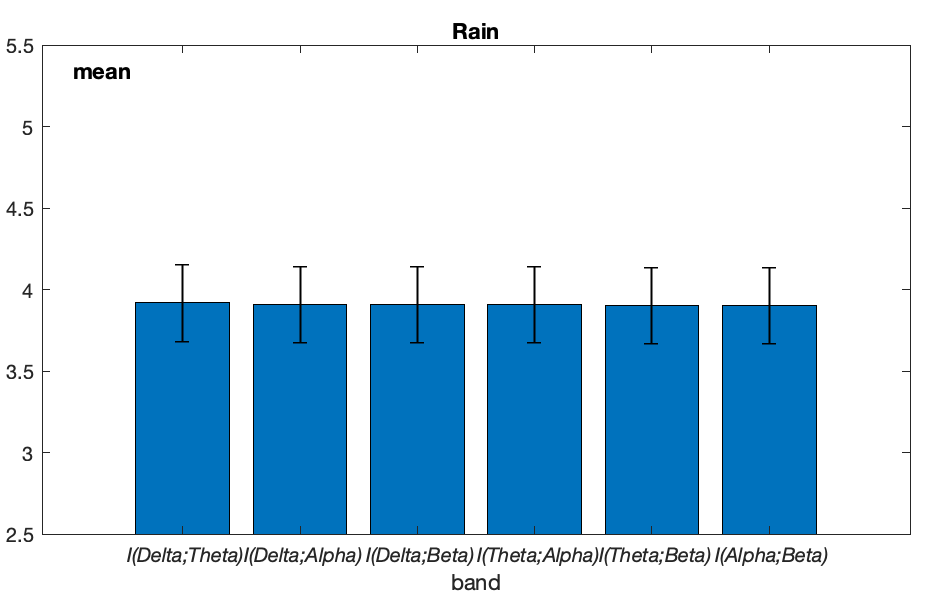

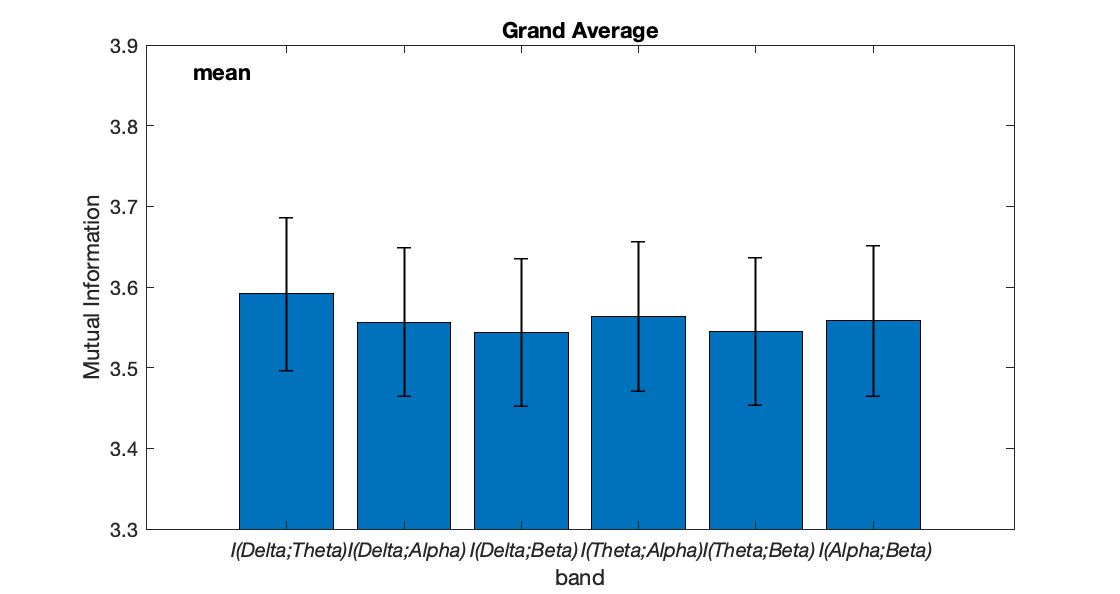
**

**
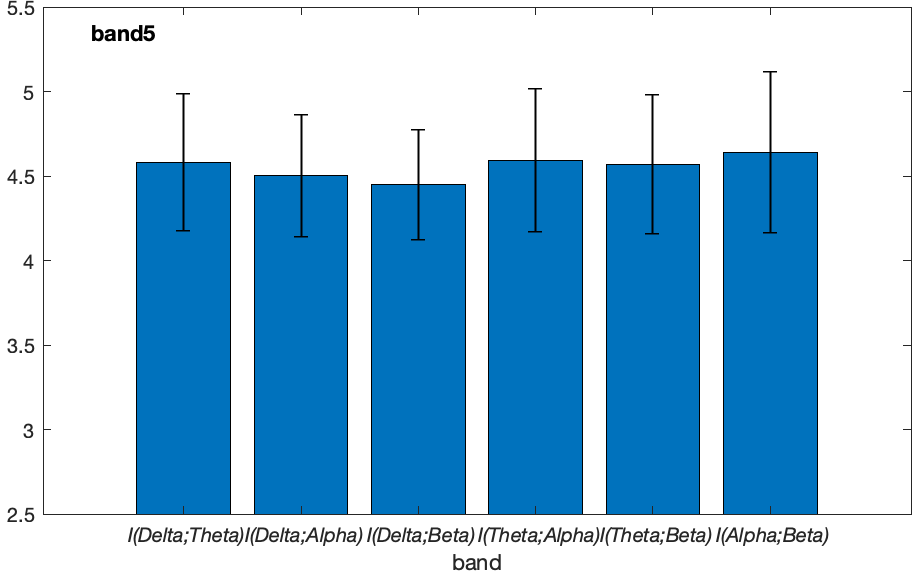

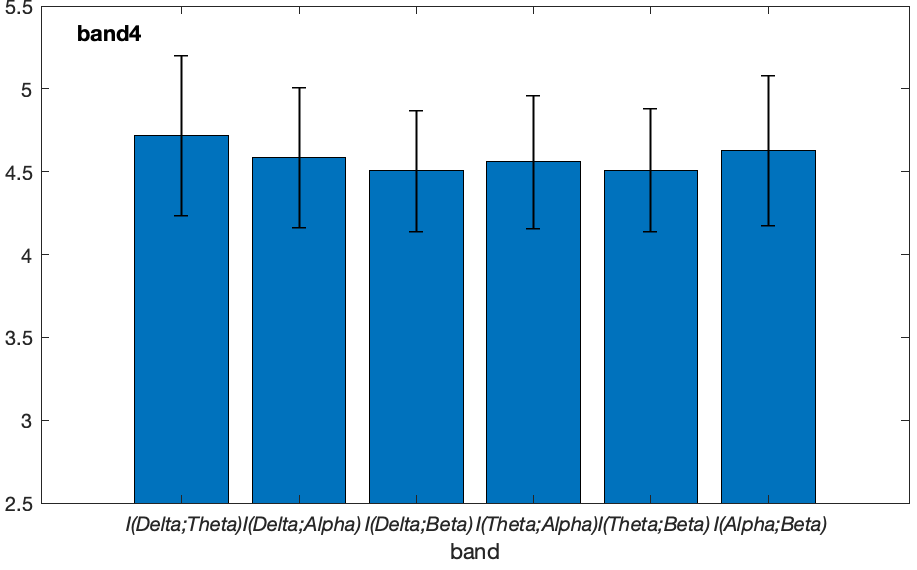

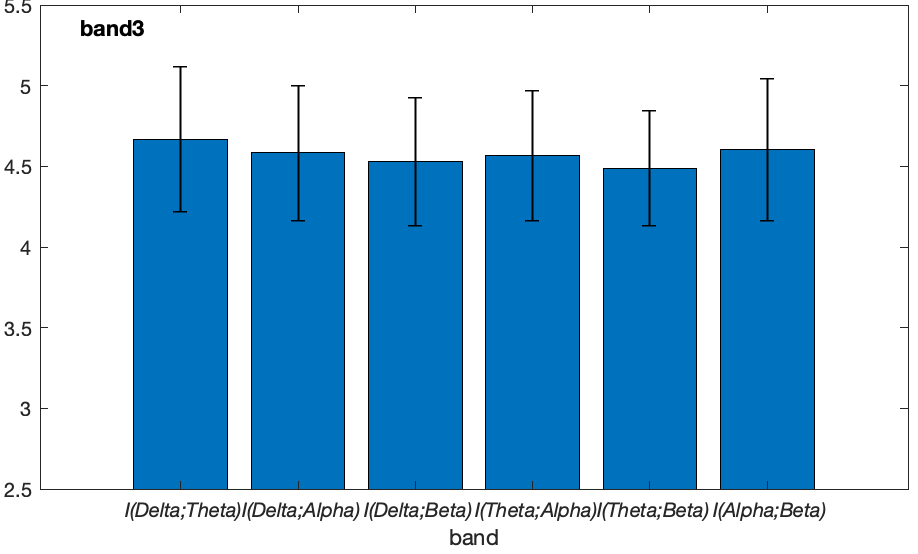

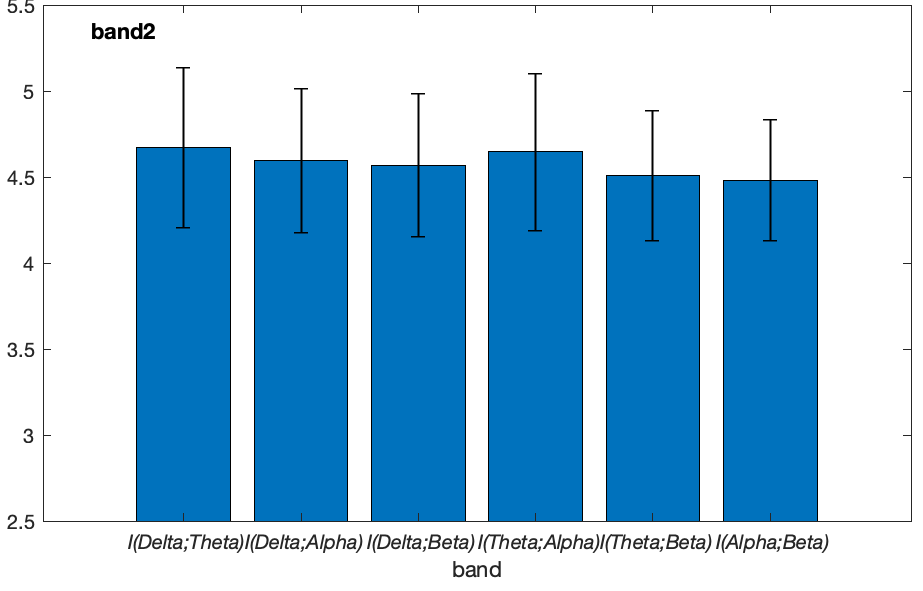

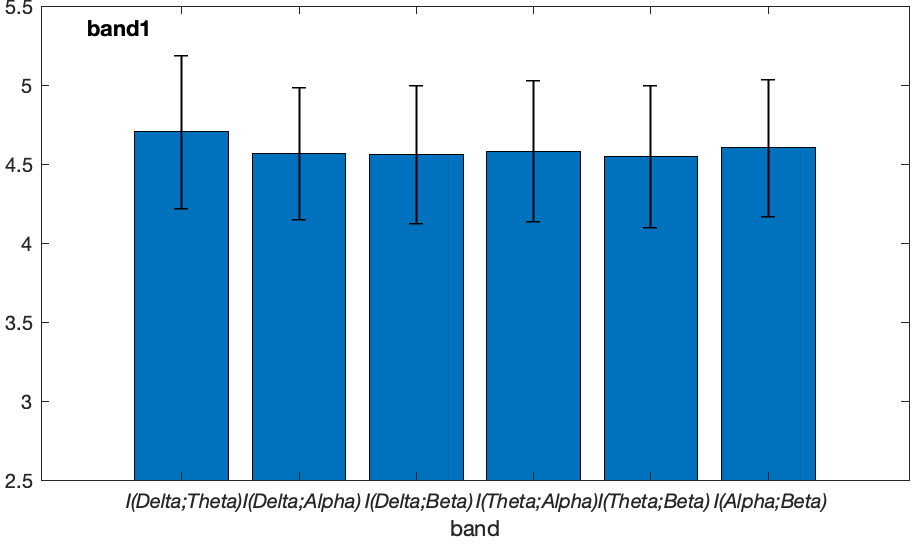

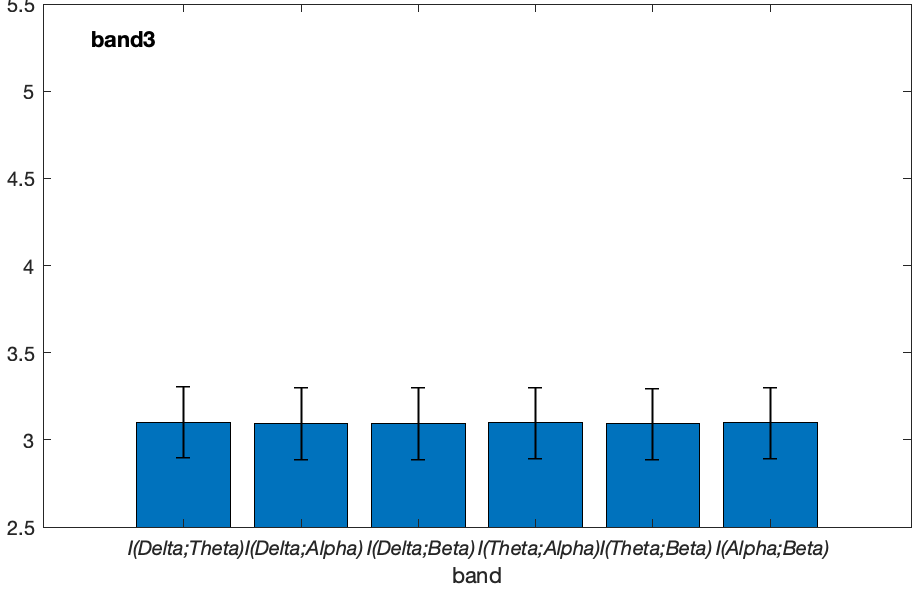

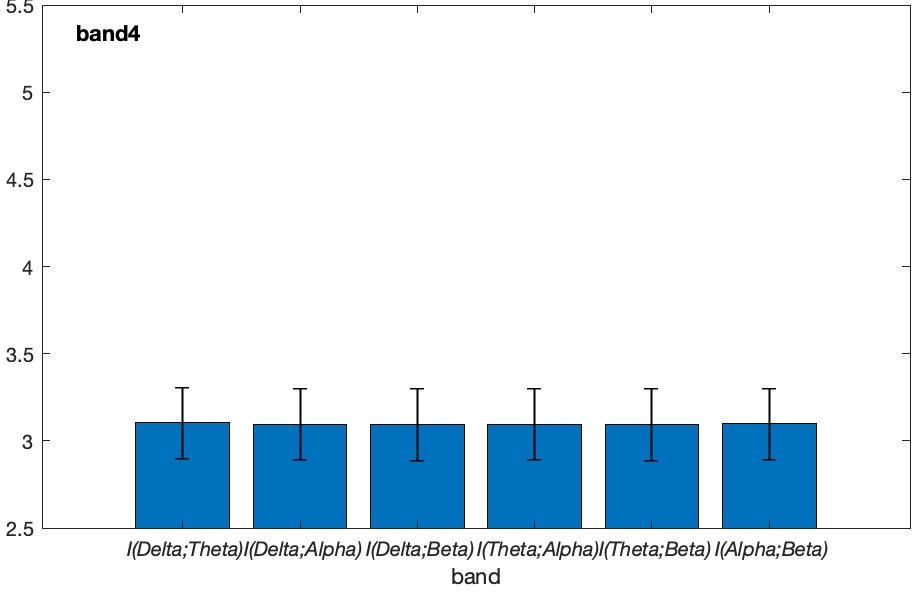

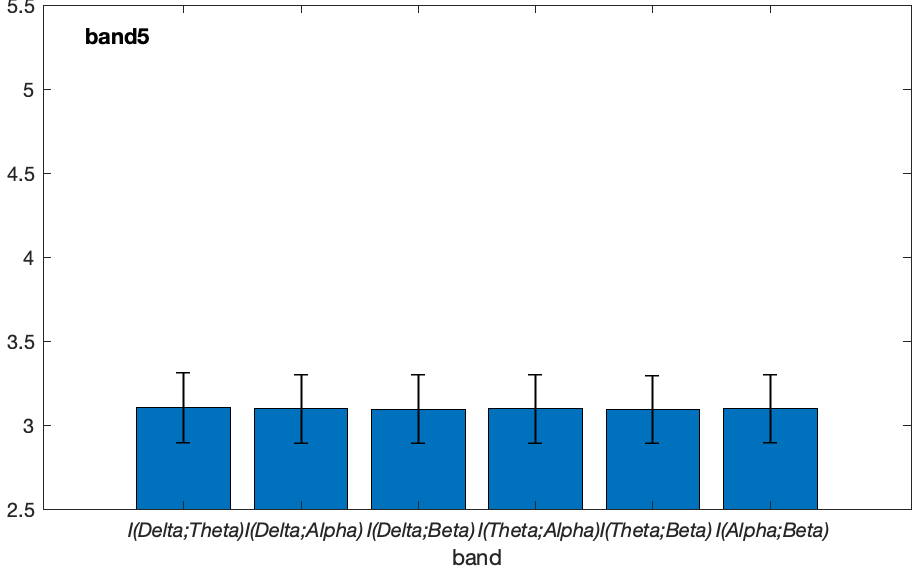

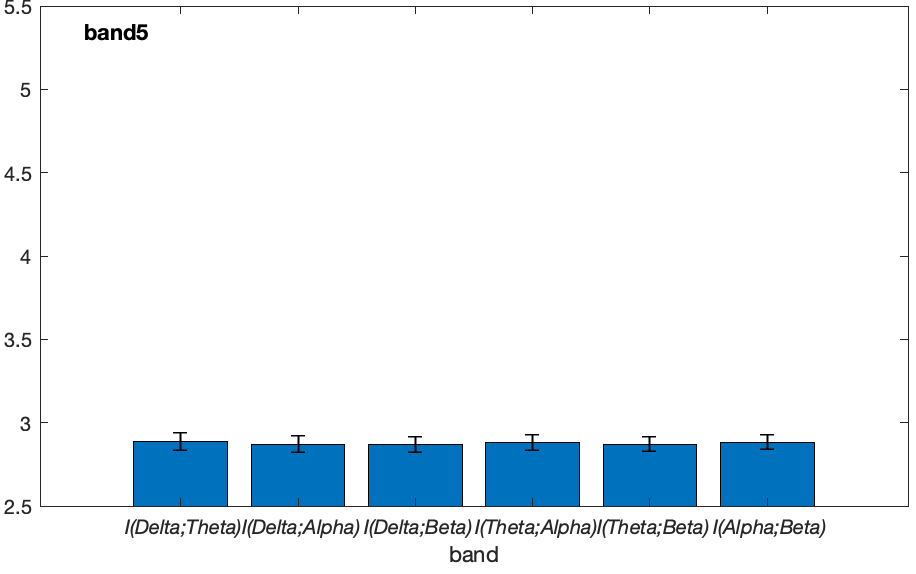

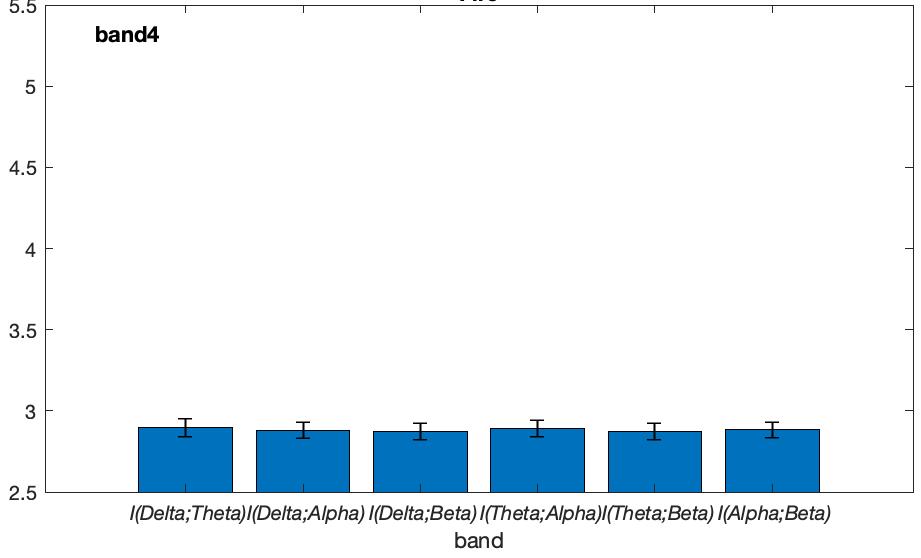

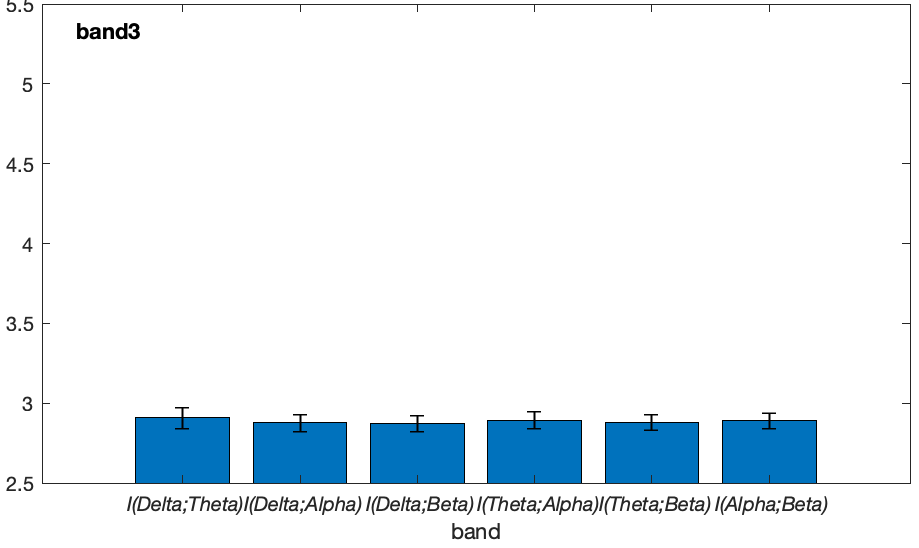

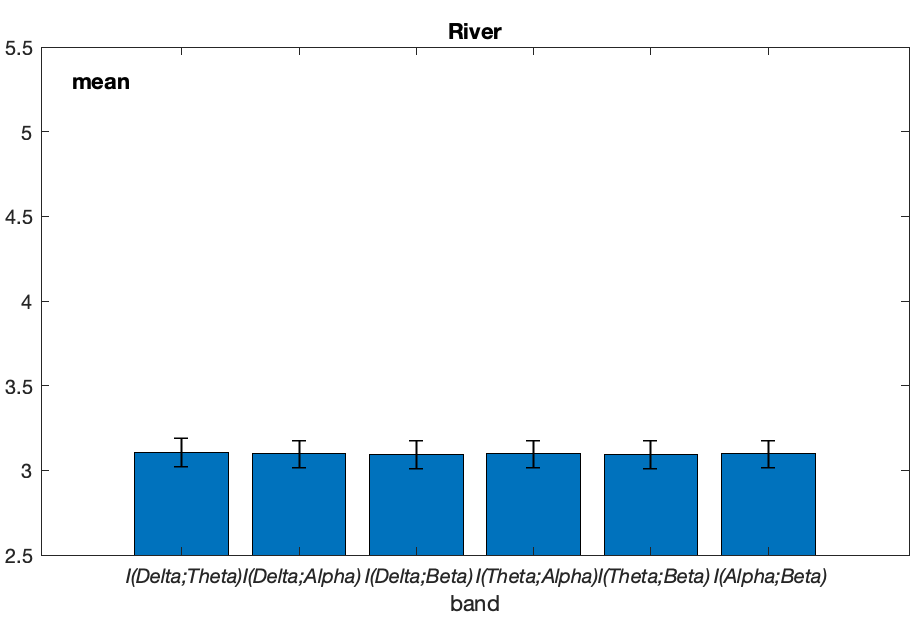

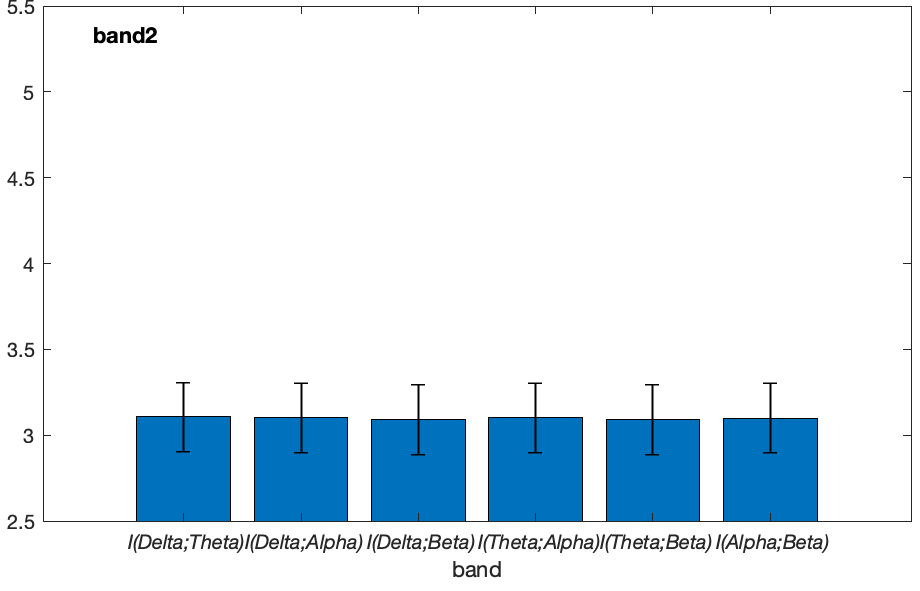

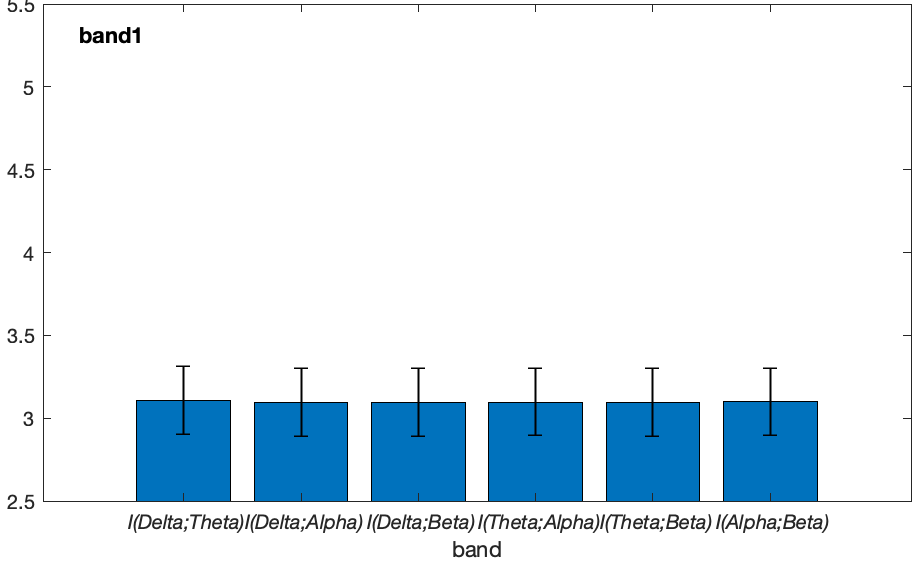

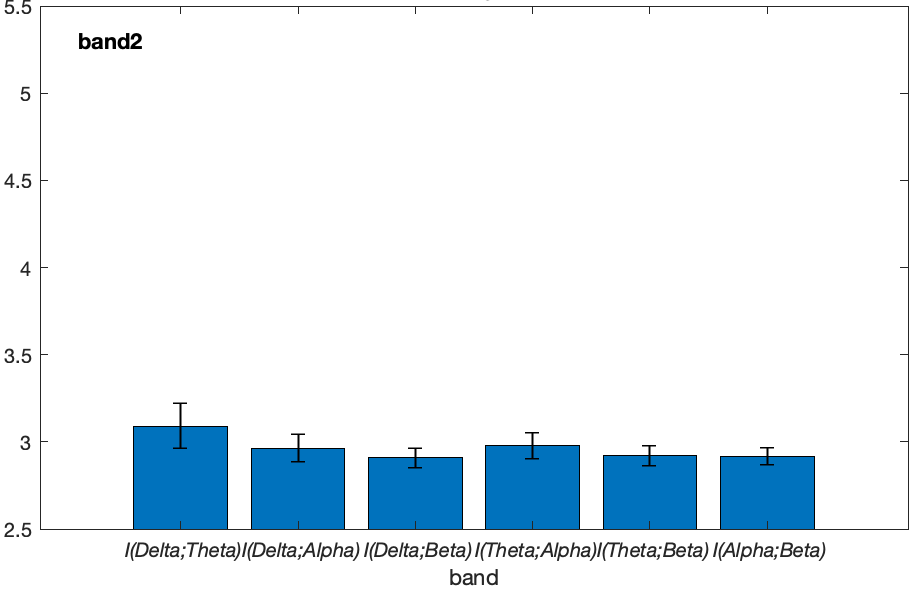

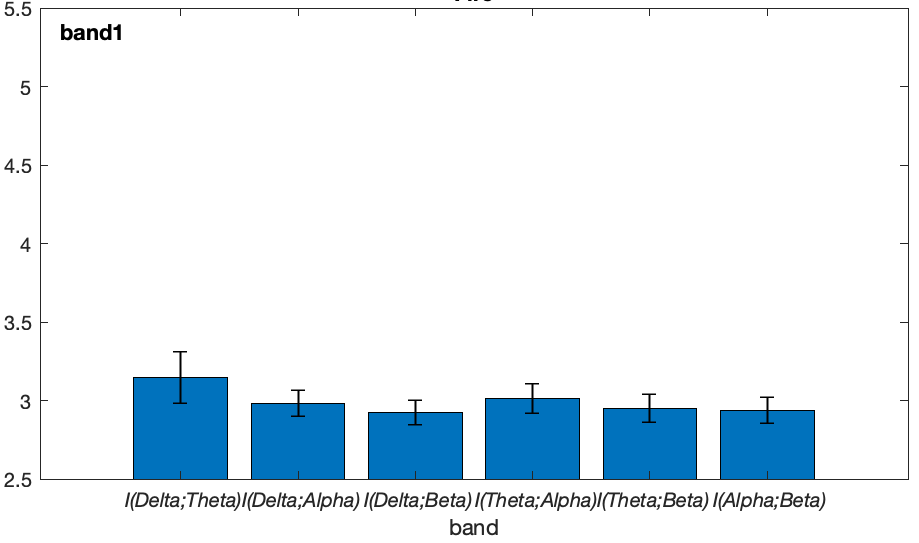

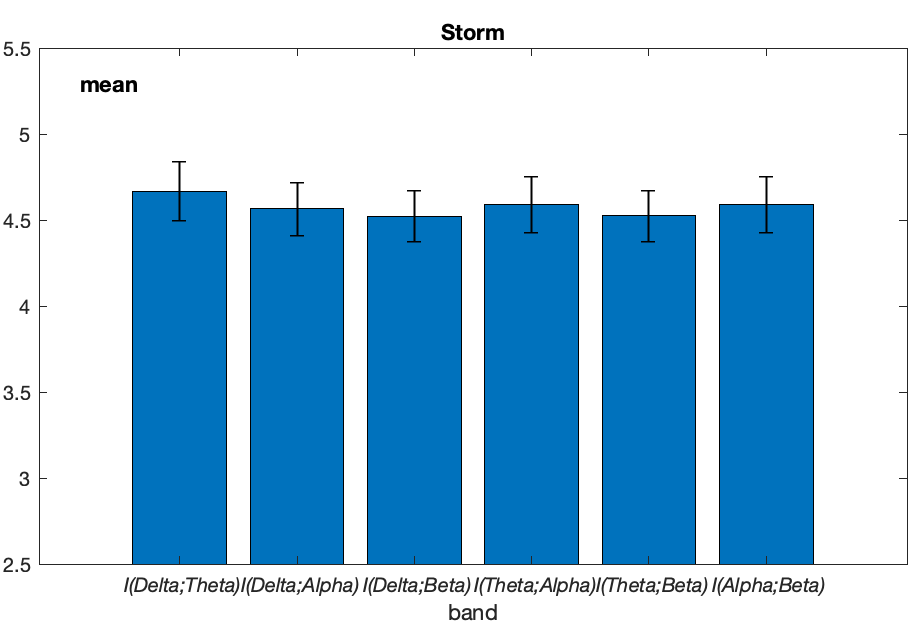

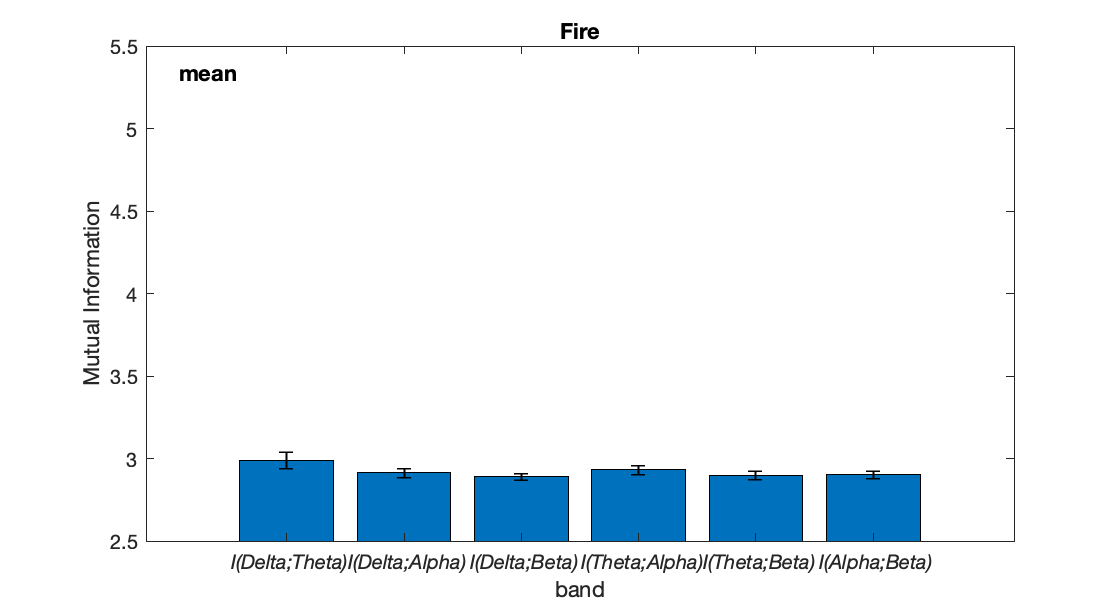
**

**Figure b**. Mutual information of nature sounds in S-AMPH model

**Mutual Information Between Adjacent Oscillatory Bands in PAD model**

Figure **c** shows the mutual information (MI) between different bands in PAD model for each genre. The MI shown here are broadly similar across the 10 genres: adjacent bands are predictable each other compared with non-adjacent band. Figure **d** shows the MI of non-music natural sounds such as rain and wind.

**Figure c**. Mutual information between different bands in PAD model


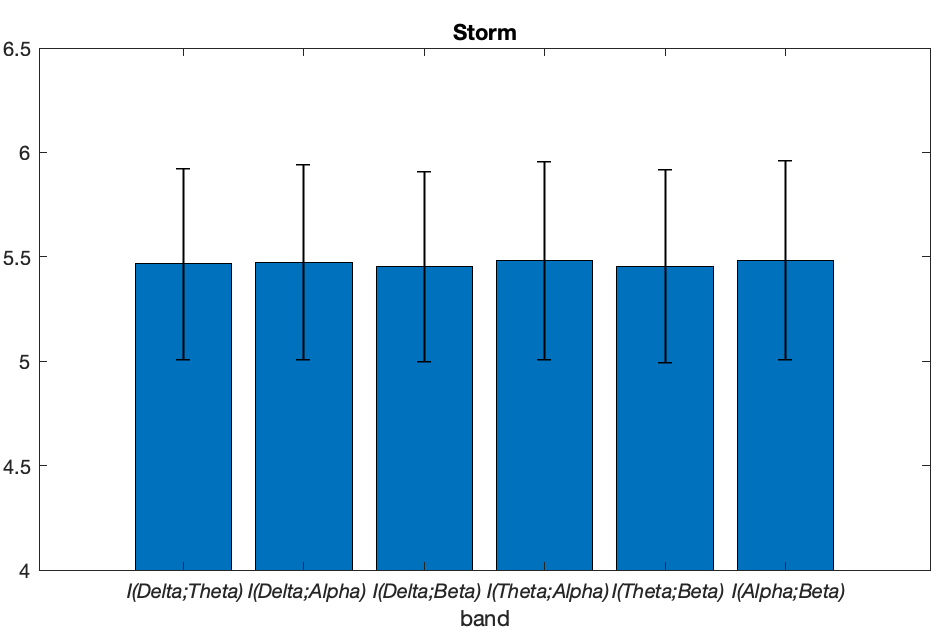

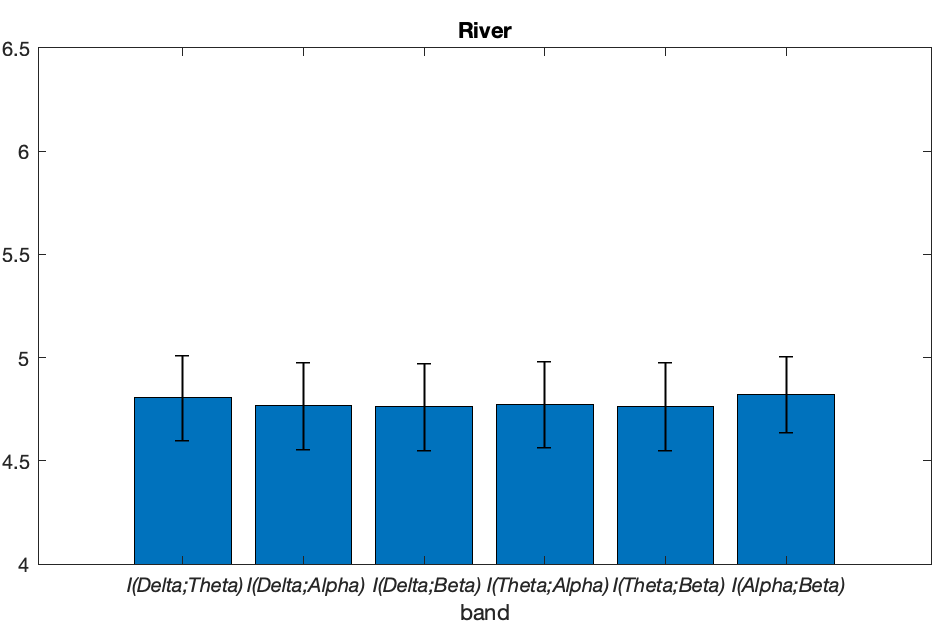

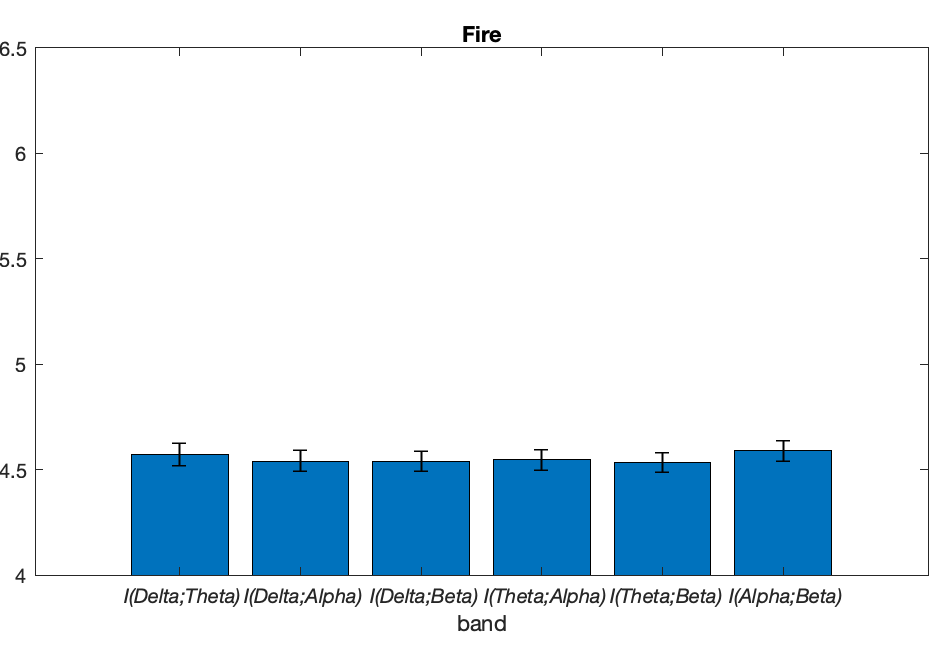

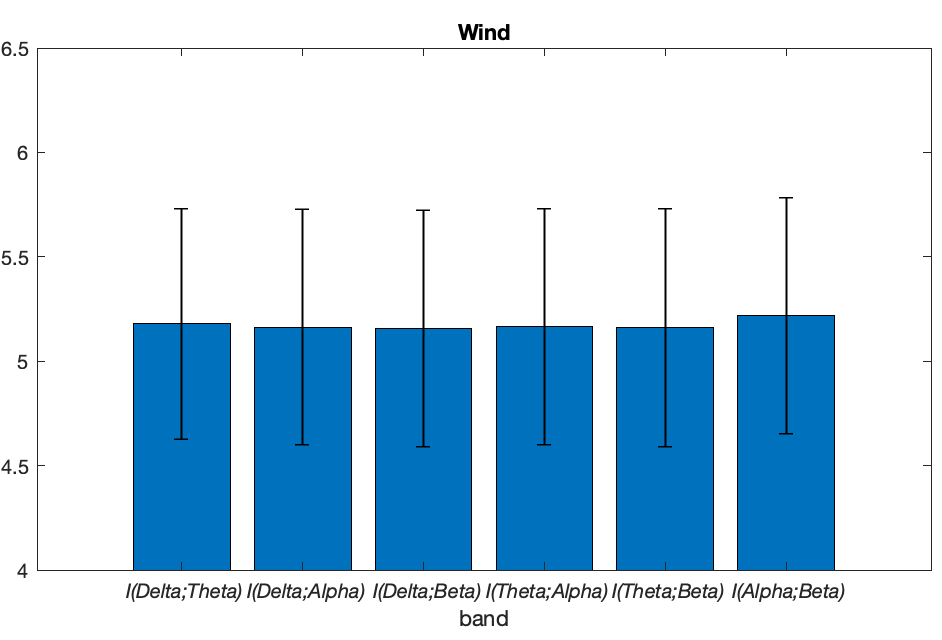

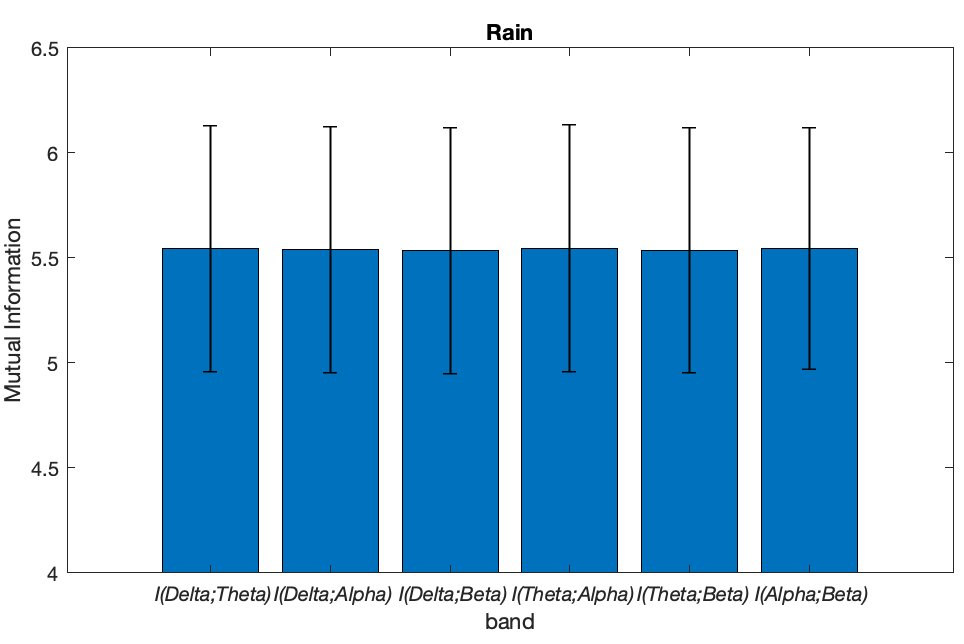

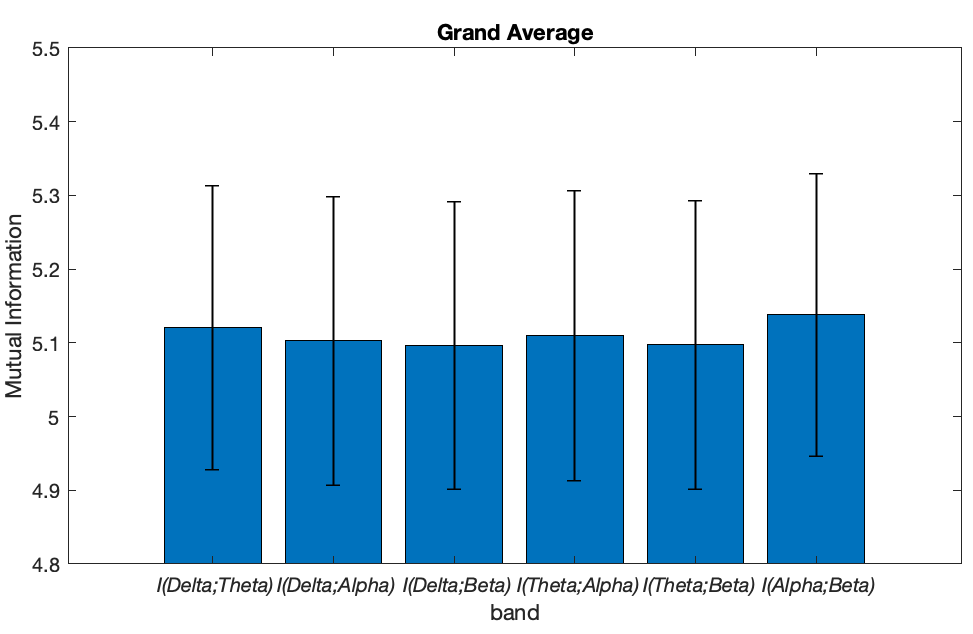


**Figure d**. Mutual information of nature sounds in PAD model
