# Supplementary material for: Hierarchical amplitude modulation structures and rhythm patterns: Comparing Western musical genres, song, and nature sounds to Babytalk
Source: PLoS One. 2022 Oct 14;17(10):e0275631. doi: 10.1371/journal.pone.0275631 (PMC9565671; doi:10.1371/journal.pone.0275631)
Supplement: Attachment — Submitted filename: Response_to_Reviewers.docx [file pone.0275631.s008.docx]

**RESPONSE TO REVIEWERS 2:**

I am extremely grateful for the Reviewers’ insightful comments on our manuscript. We have studied the Reviewers’ comments carefully and have made the necessary corrections in our paper. We believe that the comments have helped us significantly improve and refine the manuscript. Our responses to the Reviewers’ comments are as follows.

**Comment 1:**

In the introduction, the authors indicate that their modeling approach is “conceptually distinct from … models of pulse perception based on neural resonance (Large et al., 2019), oscillatory models of auditory attention based on dynamic attending”. In the discussion section, however, the authors discuss the convergence of their modelling approach with neural resonance theory. Please clarify. Also it is not clear why it is presented as being distinct from dynamic attending models as these also include multiple oscillators that entrain to the stimulus and influence processing, and also temporal sampling framework has been presented in link with dynamic attending (e.g., Goswami, 2011). Please clarify.

**Response:** We thank the reviewers for the pertinent comments. This made us reflect that our modelling is theoretically rather than conceptually distinct from other theories, while sharing some conceptual similarities with other approaches. Hence we reworded some of the sentences in the Introduction section and the Discussion section. The difference from dynamic attending theory is that DAT hypothesised multiple oscillators based on behavioural findings with attention tasks, rather than identifying a hierarchy of oscillators related to physical variations in stimuli. Here we specify the key oscillators in musical rhythm and the expected AM hierarchy *a priori,* based on our prior computational modelling of acoustic rhythm in language.

Introduction (pp. 15-16)

Note finally that our modelling approach is theoretically distinct from models that seek to identify the tactus or beat markers in singing (Coath et al., 2010), models of pulse perception based on neural resonance (Large et al., 2019), oscillatory models of auditory attention based on dynamic attending (Large & Jones, 1999), and models of temporal hierarchical structure based on the Allan Factor approach (Falk & Kello, 2017; Kello et al., 2017). Ours is the only modelling approach to analyze the modulation structure of the amplitude envelope and further to make specific *a priori* predictions concerning expected key temporal AM rates and key hierarchical AM phase relations related to the perception of musical rhythm structure and the parsing of musical units. We predict that the phase dependency between bands of AMs centred on ~2 Hz and ~ 5 Hz will relate to musical rhythm across different genres, and that music will show similar hierarchical AM structures in predictable spectral bandings to IDS, structures that can provide a perceptual basis for perceiving musical notes and musical phrasing. The amplitude envelope is recognized as core to speech processing by speech engineers (Greenberg, 2006). Our modelling decomposes the amplitude envelope of music instead of speech and then relates the resulting AM bands and their phase relationships to individual musical units. In principle, this approach provides a novel acoustic perspective on musical rhythm, motivated by our prior novel acoustic analyses of Babytalk.

Discussion (p. 51)

“The modelling presented here also converges conceptually with past studies designed to detect pulse based on neural resonance theory (Large et al., 2019).

**Comment 2:**

- Page 23: “The methodologies were based on a previous study by Leong and Goswami (2015).” Please clarify whether these were adapted (and “based on”/inspired by?) or whether the same implementations (i.e., same parameters, steps, etc.) were used here as in Leong and Goswami (2015). Otherwise, please indicate what was changed (and why).

**Response:** Thank you for the comment. This study used the same methodologies as a previous study by Leong and Goswami (2015). We now stated it in the Methods section as follows.

“This study used the same methodologies and parameters as a previous study based on CDS by Leong and Goswami (2015) (for wiki, please see https://www.cne.psychol.cam.ac.uk).” (p. 23).

**Comment 3:**

- Thanks also for adding the various appendices for further information. Regarding appendix S1, could you clarify also the average duration across the different musical pieces used? I guess “Duration (minutes)” currently just indicates the total duration of all pieces together? Or are all pieces listed underneath played in their entirety ? (no excerpts chosen). Thanks.

**Response:** I thank the reviewers for the pertinent comments. All pieces listed underneath are played in their entirety. As suggested, we described the average duration as well in the S1 Appendix, as follows.

***1) Single instrument materials***

| Instrument | Representative  Composers | Duration (minutes) | Average daution (munutes) | # pieces |
| --- | --- | --- | --- | --- |
| Piano | Beethoven, Mozart | 230 | 3.97 | 58 |
| Cello | Bach,  Ysaÿe | 173.8 | 3.78 | 46 |
| Bass | Bach | 60.9 | 3.38 | 18 |
| Viola | Bach, Hindemith | 320.8 | 4.65 | 69 |
| Violin | Bach,  Ysaÿe | 206.5 | 4.39 | 47 |
| Guitar | Bach,  Sanz | 218.4 | 3.36 | 65 |

***2) Ensemble recordings***

| Genre | Composers or  performers | Duration  (minutes) | Average daution (munutes) | # pieces |
| --- | --- | --- | --- | --- |
| Symphony | Bach, Mozart, Beethoven | 546.3 | 7.19 | 76 |
| Jazz | Miles Davis, Dave Brubeck | 212.2 | 5.89 | 36 |
| Rock | The Beatles, U2 | 226.6 | 3.28 | 69 |
| Children’s song (English) | various | 104.0 | 2.67 | 39 |
